# Epidemiology, Environmental Risks, Virulence, and Resistance Determinants of *Klebsiella pneumoniae* From Dairy Cows in Hubei, China

**DOI:** 10.3389/fmicb.2022.858799

**Published:** 2022-05-04

**Authors:** Xiangyun Wu, Jiayi Liu, Jiawei Feng, Muhammad Abu Bakr Shabbir, Yali Feng, Rui Guo, Meifang Zhou, Sulin Hou, Guiqiang Wang, Haihong Hao, Guyue Cheng, Yulian Wang

**Affiliations:** ^1^MOA Laboratory for Risk Assessment of Quality and Safety of Livestock and Poultry Products, Huazhong Agricultural University, Wuhan, China; ^2^Institute of Microbiology, University of Veterinary and Animal Sciences, Lahore, Pakistan; ^3^Hubei Livestock and Poultry Breeding Centre, Wuhan, China; ^4^National Reference Laboratory of Veterinary Drug Residues (HZAU) and MOA Key Laboratory for Detection of Veterinary Drug Residues, Huazhong Agricultural University, Wuhan, China

**Keywords:** *Klebsiella pneumoniae*, cow mastitis, antimicrobial resistance, virulence gene, *wzi* gene sequencing, MLST

## Abstract

*Klebsiella pneumoniae* (*K. pneumoniae*) is an opportunistic pathogen, which causes serious infections in humans and animals. To investigate the antimicrobial resistance pattern and virulence profile of *K. pneumoniae*, a total of 887 samples were collected from both the healthy and mastitis cows and the bedding, feed, feces, air, drinking water, spraying water, washing water, and milk cup swabs from five dairy farms in Hubei, China, during 2019 and 2020. *K. pneumoniae* was isolated and identified using PCR of the *khe* and 16S rDNA sequencing. A genotypic characterization was performed for *K. pneumoniae* isolates using *wzi* typing *and* multilocus sequence typing (MLST). Antimicrobial resistances were confirmed using broth microdilution against 17 antimicrobial agents and resistance and virulence genes were determined by PCR. The prevalence of *K. pneumoniae* was 26.94% (239/887) distributed in 101 *wzi* allele types (199/239, 83.26%) and 100 sequence types (STs) (209/239, 87.45%), including 5 new *wzi* allele type and 25 new STs. Phylogenetic analysis showed that *K. pneumoniae* isolated from milk, nipple swab, feed, and feces is classified in the same clone complex. By comparing with the PubMLST database, at least 67 STs have the risk of spreading in different species and regions. Interestingly, 60 STs have been isolated from humans. The isolates were highly sensitive to meropenem and colistin, but resistant to ampicillin (100%), sulfisoxazole (94.56%), cephalothin (47.28%), streptomycin (30.13%), and so on. Noteworthy, multidrug-resistant (MDR) rate was found to be 43.93% in this study. By PCR, 30 of 68 antimicrobial resistance (AMR) genes were identified; the prevalence rate of *blaTEM*, *blaSHV*, *strA*, *strB*, *aadA1*, and *aac(6′)-Ib-cr* was more than 50%. Eleven *CTX-M*-producing *K. pneumoniae* were found. The detection rate of *fimH*, *mrkD*, *uge*, *wabG*, *entB*, *iutA*, *iroN*, and *ureA* was over 85%. This study reinforces the epidemiological importance of *K. pneumoniae* in food-producing animals in Hubei. The emergence and spread of environmental MDR *K. pneumoniae* may pose a potential threat to food safety and public health.

## Introduction

Cow mastitis is considered to be one of the most common and frequent diseases in dairy herds. This disease only affects the estrus and pregnancy of dairy cows, resulting in the decline of milk production and quality, but also increases treatment costs and causes high economic losses in dairy industries worldwide (Käppeli et al., 2019). The global economic losses per year due to mastitis amount to $35 billion and for the United States dairy industry $2 billion per year (Sathiyabarathi et al., 2016; Li et al., 2019). Besides, mastitis can pose a threat to human and animal health *via* the transfer of antimicrobial resistance bacteria and food poisoning (Li et al., 2019). *Klebsiella pneumoniae* (*K. pneumoniae*) is one of the main environmental pathogens causing mastitis, as well as a pathogen of zoonotic conditions that can cause a series of serious infections such as respiratory tract infections, urinary tract infections, soft-tissue infections, and bloodstream infections (Lee et al., 2017; Massé et al., 2020).

Virulence factors play an important role in the pathogenic mechanism of *K. pneumoniae*. Capsular, iron carriers, pili, and lipopolysaccharide (LPS) have been widely demonstrated to be involved in the adhesion, invasion, and growth of *K. pneumoniae* (Shon et al., 2014; Cheng et al., 2020). Capsular can prevent *K. pneumoniae* from being recognized by the host immune system through some immune escape mechanisms such as antiphagocytosis, inhibition of early inflammatory response, neutralization of antimicrobial peptides to reduce the body’s immune response, and inhibition of dendritic cell maturation (Paczosa and Mecsas, 2016). These bacteria can absorb the iron of the host *via* four siderophores such as aerobactin, salmochelin, enterobactin and yersiniabactin for metabolism and enhance the virulence to cause infection (Wang G. et al., 2020). Two common types of fimbriae are found in *K. pneumoniae*: type 1 (*fim*) and type 3 (*mrk*) fimbriae. Type 1 fimbriae can enhance virulence by adhering to mucosal or epithelial surfaces and type 3 fimbriae adhere to the cell surface and promote biofilm formation (Schroll et al., 2010). Lipid A, a component of lipopolysaccharide, reduces the inflammatory response during *K. pneumoniae* infection and prevents the bactericidal effect of cationic antimicrobial peptides. O antigen is the outermost subunit of LPS, which eliminates the lysis of bacteria by the complement membrane attack complex (Paczosa and Mecsas, 2016).

Previous studies have shown that mucoviscosity-associated gene (*magA*), uridine diphosphate galactose 4 epimerase encoding gene (*uge*), regulator of the mucoid phenotype (*rmpA*), iron uptake system gene (*kfu, aerobactin*), and K1 and K2 capsule serotypes are important virulence genes in invasive *K. pneumoniae* strains causing mastitis (Osman et al., 2014; Massé et al., 2020). According to virulence characteristics, *K. pneumoniae* can be divided into classic *Klebsiella pneumoniae* (cKP) and hypervirulence *Klebsiella pneumoniae* (hvKP). Most *K. pneumoniae* infections in the world are cKP infections, but cKP can evolve into hvKP by obtaining virulence factors (such as plasmids) (Wyres et al., 2020). Studies have shown that the genes *peg-344*, *iroB*, *iucA*, *_*p*_rmpA*, and *_*p*_rmpA2* can distinguish cKP and hvKP with high accuracy (Harada and Doia, 2018; Russo et al., 2018). Some scholars have found that K1 and K2 capsule serotype is closely related to high virulence (Follador et al., 2016; Guo et al., 2017). Since hvKP was first detected in Taiwan in 1986, the number of clinical infection cases of the bacteria has gradually increased and Wuhan was the city with the highest hvKP prevalence (73.9%) reported among 230 *K. pneumoniae* isolates from 10 cities in China (Liu et al., 1986, 2014; Zhang et al., 2016). More worryingly, with the inappropriate use of antibiotics, multidrug resistant (MDR) has been increased all over the world that is considered as a public health threat. Several recent investigations reported the emergence of multidrug-resistant bacterial pathogens from different origins, including humans, birds, cattle, and fish that increase the need for new potent and safe antimicrobial agents (Algammal et al., 2020a,b, 2021). Epidemics and outbreaks of multidrug-resistant *K. pneumoniae* have also been reported around the world (Gu et al., 2018; Alanezi et al., 2022; Arteaga-Livias et al., 2022). The worldwide spread of extended-spectrum β-lactamase (ESBL)-producing *K. pneumoniae* remains a critical concern for therapies against multidrug-resistant bacteria (Chong et al., 2018). “Superbug” derived from the combination of virulence and resistance of *K. pneumoniae* will bring great challenges to clinical anti-infective treatment. In addition, hundreds of mobile antimicrobial resistance (AMR) genes have been found in *K. pneumoniae* (Navon-Venezia et al., 2017). *K. pneumoniae* is considered to be a major transporter of resistance genes from environmental sources to clinically important bacteria and some isolates can carry acquired AMR genes or plasmid to move between environmental, human, and animal (Wyres and Holt, 2018).

As the most important measures for disease prevention and control, finding the source of infection and cutting off the transmission route are to block the transmission of pathogens from infected animals/people to susceptible animals/people (Chen et al., 2021). Genotyping technology is mainly used to understand the transmission route of pathogens and trace the source of infection. Multilocus sequence typing (MLST) is widely used because of its good typing ability, comparability, high reproducibility, high throughput, and convenient data sharing (Pérez-Losada et al., 2013). The sequence types (STs) of *K. pneumoniae* reported to be important and prevalent globally include ST11, ST15, ST23, ST258, ST395, ST512, and so on (Zhang et al., 2019; Gato et al., 2020; Zhou et al., 2020; Di Pilato et al., 2021). Currently, the host and regional epidemiological characteristics of *K. pneumoniae* in various regions are still unclear and surveillance data on resistance and virulence of *K. pneumoniae* in the cow are very scarce. This study aimed to investigate the prevalence, antimicrobial resistance pattern, resistance genes, and virulence genes of *K. pneumoniae* isolated from dairy cows in Hubei, China. *K. pneumoniae* from milk and environmental samples was genotyped using PCR to determine genetic diversity and explore potential reservoirs and transmission.

## Materials and Methods

### Farms and Sample Collection

Samples were collected from five commercial dairy farms in Hubei during 2019 and 2020. The number of lactating cows in farms A, B, C, D, and E are 202, 300, 400, 1,200, and 287, respectively. Five farms participated in monthly Dairy Herd Improvement (DHI). Lactating cows in five farms were milked 2 times/day in milking parlors. The clinical symptoms of cows with clinical mastitis (CM) are elevated body temperature, redness, and pain in the udder. The judgment of subclinical mastitis (SCM) refers to the Chinese agricultural industry standard NY/T 2692-2015 (National standards of the people’s Republic of China, 2015) [the number of somatic cell count (SCC) is > 500,000 cells/ml and no visible pathological changes]. Daily care of cows in five farms was provided by the veterinarian; the farms routinely used different antibacterial agents such as ceftiofur, amoxicillin/clavulanic acid, lincomycin, florfenicol, kanamycin, and gentamicin for regular prophylactic and treatment protocols. The sample collection includes animal samples (nipple milk, nipple skin swabs, anal swabs from healthy cows, CM cows, and SCM cows) and environmental samples (bedding, feed, feces, air, drinking water, spraying water, washing water, and milk cup swabs). Lactating cows of parity 3rd to 4th were selected for sample collection. Briefly, milk samples were collected from lactating cows by the milkers after premilking disinfection and the first 3 streams of milk were discarded. Commercial liquid delivery mediums (Haibo Biotech, Qingdao, China) were used to collect nipple skin swabs, anal swabs, and milk cup swabs. All the nipples of each cow were rubbed on the skin with swabs 4 to 5 times from the root to the end of teat after milking. For the anal swab, the swab was inserted into the anus of the cow and rotated for 2 to 3 circles. The swab of the milk cup is collected in milking parlors (5 per farm). Feed, bedding, and feces samples were stored in sterile sampling bags and air samples were collected by natural sedimentation method [suspended sterile sampling tube with 15 ml Trypticase Soy Broth (Haibo Biotech, Qingdao, China) on the column in the barn for 15 to 30 min]. Three sampling points were evenly selected in the barn and the interval between sampling points was not less than 5 m and then mix the samples with equal weight. All the samples were stored at 4°C and transported to the laboratory for bacterial culturing and identification within 24 h. SCC of milk sample was analyzed by DHI Center of Livestock and Poultry Breeding Centre in Hubei.

### Isolation and Identification of *Klebsiella pneumoniae*

All the samples were inoculated in 5 ml Trypticase Soy Broth (Haibo Biotech, Qingdao, China) and incubated at 37°C for 18 to 24 h. A loopful of broth culture was streaked onto Columbia Blood Agar and MacConkey Inositol Adonitol Carbenicillin (MIAC) agar (Haibo Biotech, Qingdao, China) and incubated at 37°C for 12 to 18 h. A single pink, slimy, and non-hemolytic suspected colony was picked and purified on MIAC agar (Gao et al., 2010). Purified bacteria were selected for biochemical identification and PCR testing. Gram staining was performed with the Gram Kit (Nanjing Jiancheng Bioengineering Institute, Nanjing, China). Biochemical identification tubes (Haibo Biotech, Qingdao, China) were used for motility tests, indole tests, urease tests, oxidase experiments, and citrate utilization tests (Massé et al., 2020). Further identification and confirmation of the isolates were carried out using PCR of *khe* and *16s rDNA* gene (He et al., 2016; Cheng et al., 2021). Positive clones were sequenced by the Tsingke Biotechnology Corporation Ltd. (Wuhan, China). *K. pneumoniae* ATCC 700603 (*khe* positive) and *Escherichia coli* ATCC 25922 (*khe* negative) were used as control strains.

### Deoxyribonucleic Acid Extraction

Genomic DNA of *K. pneumoniae* was extracted by boiling method (Kuang, 2015). 3 ml suspended plaque samples were incubated for 15 to 20 min at 99°C. Subsequently, the supernatants were immediately transferred to −20°C for 15 min and centrifuged at 12,000 rpm/min for 15 min at 4°C. The supernatants were diluted by sterile water (1:10) and kept at −20°C for PCR testing.

### Capsular Serotyping and Multilocus Sequence Typing

Capsular polysaccharide serotypes were determined by *wzi* gene sequencing (Brisse et al., 2013). MLST detection method refers to the *K. pneumoniae* MLST online database^1^. The seven housekeeping genes (*gapA*, *infB*, *mdh*, *phoE*, *pgi*, *rpoB*, and *tonB*) were amplified by PCR. All the positive products were sequenced by Sanger and sequences were submitted to the website^2^ to get the *wzi* allele types and the subtypes of each housekeeping gene of MLST. Submit the allele profile to the MLST website^3^ to get ST. *wzi* alleles and STs that had not been previously described were submitted to the curator of the PubMLST database and were assigned new designations. Sanger sequencing was completed by Tsingke Biotechnology Corporation Ltd. (Wuhan, China).

### Phylogenetic Analysis and Comparison of *Klebsiella pneumoniae* From Different Sources and Countries in the Multilocus Sequence Typing Database

The ST phylogeny tree (Tamura 3-parameter model) was constructed using the maximum likelihood method in the MEGA-X software and was modified visually with interactive tree of life (iTOL)^4^. The phylogenetic tree is tested by the bootstrap method and the number of tests is 1,000 times. The information of 726 *K. pneumoniae* isolates from different countries and 724 *K. pneumoniae* isolates from different hosts was downloaded from the PubMLST database (the same 100 STs as in this study). Minimum spanning tree (MST) was constructed by Bionumerics 8.1.

### Detection of Virulence Genes

All the 239 *K. pneumoniae* were evaluated for the presence of 23 known virulence genes, including transporter (*peg344*), fimbriae synthesis-related gene (*fimH, mrkD*), lipopolysaccharide-related gene (*uge*, *wabG*), capsular polysaccharide synthesis and synthesis regulation-related gene (*wcaG*, *_*c*_rmpA*, *_*p*_rmpA*, *_*p*_rmpA2*, and *magA*), iron uptake system (*iroB*, *iroN*, *aerobactin*, *iutA*, *irp2*, *iucA*, *ybtA*, *kfu*, and *entB*), urease-related gene (*allS*, *ureA*), tellurite resistance gene (*terB*), and hemolysin (*hly*) by PCR (Supplementary Table 1).

### Antimicrobial Susceptibility Testing

The susceptibility of *K. pneumoniae* isolates to 17 antimicrobials, which are commonly used on dairy farms and become clinically important broad-spectrum antimicrobials, was determined using the microbroth dilution method recommended by the Clinical and Laboratory Standardization Institute [Clinical and Laboratory Standards Institute [CLSI] (2021)], including penicillins: ampicillin (AMP); β-lactam/β-lactamase-inhibitor combinations: amoxicillin/clavulanic acid (AMC); cephalosporins: cephalothin (CEP), ceftiofur (EFT), and ceftriaxone (CRO); carbapenems: meropenem (MEM); aminoglycosides: streptomycin (STR), kanamycin (KAN), and gentamicin (GEN); tetracyclines: tetracycline (TET) and doxycycline (DOX); amphenicols: florfenicol (FFC) and polymyxins: colistin (CT), rifamycins: rifaximin (RFX), fluoroquinolones: ciprofloxacin (CIP); and sulfonamides: sulfisoxazole (SOX) and sulfamethoxazole/trimethoprim (SXT). Results were interpreted according to the CLSI M100-Ed31 and VET01S-Ed5 standards [Clinical and Laboratory Standards Institute [CLSI] (2020, 2021)]. No CLSI interpretative criteria for ceftiophene and streptomycin are currently available and the resistance breakpoint of ceftiophene and streptomycin refers to ceftiofur and kanamycin, respectively. The criteria for resistance of rifaximin were analyzed by Ecofinder software recommended by the European Committee on Antimicrobial Susceptibility Testing (EUCAST) (Turnidge et al., 2006). The phenotypic resistance patterns are categorized into MDR, extensively drug-resistant (XDR), and pandrug-resistant (PDR) as previously described by Magiorakos et al. (2012). The quality control strain is *K. pneumoniae* ATCC 700603 and *Escherichia coli* ATCC 25922.

### Detection of Antimicrobial Resistance Genes

Antimicrobial resistance genes that confer resistance to β-lactams (*bla*_*CTX–M–2*_, *bla*_*CTX–M–10*_, *bla*_*CTX–M–14*_, *bla*_*CTX–M–15*_, *bla*_*SHV*_, *bla*_*TEM*_, and *bla*_*OXA–1*_), carbapenems (*IMP*, *VIM*, *bla*_*OXA–48*_, *bla*_*OXA–181*_, *NDM*, and *KPC*), AmpCs (*MOX*, *CIT*, *DHA*, *ACC*, *EBC*, and *FOX*), aminoglycosides [*armA*, *rmtA*, *rmtB*, *rmtC*, *rmtD*, *rmtE*, *npmA*, *aadA1*, *aadA2*, *aadB*, *aacC1*, *aacC2*, *aac(3)-IV*, *aacA4*, *aphA1*, *aphA2*, *aphA6*, *strA*, and *strB*], plasmid-mediated quinolone resistance (PMQR) genes [*aac(6′)-Ib-cr*, *qnrA*, *qnrB*, *qnrC*, *qnrD*, and *qnrS*], tetracyclines [*tet*(A), *tet*(B), *tet*(C), *tet*(D), *tet*(E), and *tet*(G)], sulfonamides (*sul1*, *sul2*, and *sul3*), macrolides (*mefA*, *ereA*, *ereB*, *ermB*, *mphA*, and *mphB)*, polymyxins (*mcr-1* to *mcr-5*), and phenicols (*floR*, *cmlA*, and *Cat1*) were investigated by PCR (Supplementary Table 2). For each positive gene, some PCR products were selected and sent to Quintara Biotechnology Corporation Ltd. (Wuhan, China) for Sanger sequencing for further confirmation.

### Amplification and Sequencing of the Quinolone Resistance-Determining Regions Mutation Genes Fragments

All of ciprofloxacin-resistant *K. pneumoniae* were selected to determine the DNA sequence of quinolone resistance-determining region (QRDR) genes (*gyrA*, *gyrB*, *parC*, and *parE*) (Horii et al., 2008; Pitondo-Silva et al., 2015; Cheng et al., 2018). Three ciprofloxacin-intermediate and 7 ciprofloxacin-sensitive isolates were randomly selected as controls. Genomic DNA was extracted by the Universal Genomic DNA Kit of ComWin Biotechnology Corporation Ltd. (Beijing, China). PCR products were purified and sequenced in both the directions by Quintara Biotechnology Corporation Ltd. (Wuhan, China). The nucleotide sequences and the deduced amino acids were compared with that of *K. pneumoniae* ATCC 13883 (GenBank JOOW00000000.1) using the Bioedit (Azargun et al., 2019).

### Statistical Analyses

SPSS statistics version 26 and GraphPad Prism version 8.0.1 software were used for statistical analysis and the chi-squared test (χ^2^) was used to compare the statistical significance between the different groups. *p* < 0.05 means significant difference and *p* < 0.01 indicates extremely significant difference. Correlations were assessed by calculating the Spearman’s rank correlation coefficient (r). |r| ≥ 0.8 means high correlation, 0.5 ≤ | r| < 0.8 means moderate correlation, 0.3 ≤ | r| < 0.5 means low correlation, and | r| < 0.3 means no correlation.

## Results

### Phenotypic Characteristics of *Klebsiella pneumoniae* Isolates

*Klebsiella pneumoniae* isolates grew well on MIAC agar due to resistance to carbenicillin and gave characteristic pink colonies as a result of inositol and adonitol fermentation. On Columbia Blood Agar, the colonies are not hemolytic and tend to be viscous and stringy. *K. pneumoniae* was observed as Gram-negative bacillus under a light microscope. Besides, it showed the characteristics of non-motility, indole negativity, production of urease, negative oxidase testing, and utilization of citrate.

### Prevalence of *Klebsiella pneumoniae* in Five Dairy Farms

**FIGURE 1:**
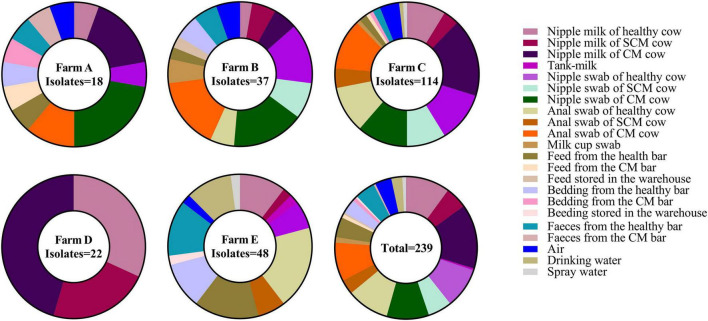
The percentage of *Klebsiella pneumoniae* (*K. pneumoniae*) in different sample types in five farms.

A total of 239 *K. pneumoniae* (26.94%) were identified in 887 samples through biochemical identification, *khe* amplification, and 16s rDNA sequencing (Figure 1 and Supplementary Table 3). The samples collected in farm C (114/338, 33.73%) showed a significantly higher *K. pneumoniae* prevalence than farm A (18/109, 16.51%), farm B (37/179, 20.67%), and farm E (48/189, 25.40%). The *K. pneumoniae* prevalence in farm D (22/72, 30.56%) was also significantly higher than farm A (18/109, 16.51%) (*p* < 0.05). There was no significant difference in the *K. pneumoniae* prevalence of nipple milk, skin swabs, and anal swabs between healthy and mastitis cows (*p* > 0.05). Besides, *K. pneumoniae* was found in a variety of environmental sources, such as milk cup swab, feed, bedding, feces, air, drinking water, and spray water and the separation rate is between 15.79 and 47.83%.

### Capsular Serotyping and Multilocus Sequence Typing

**FIGURE 2:**
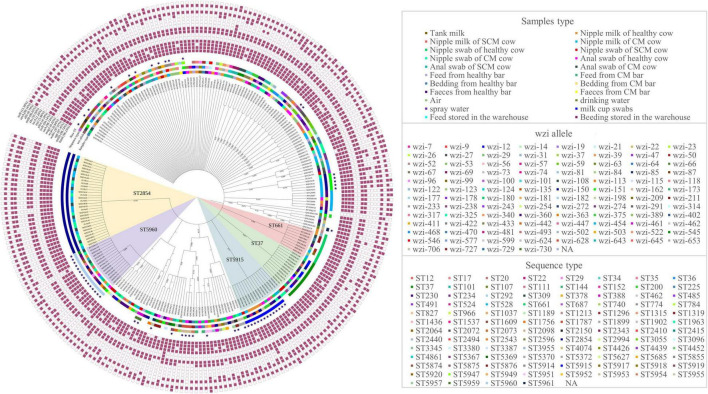
Sequence type (ST) phylogenetic tree, *wzi* allele, and detection of virulence of 239 *K. pneumoniae*. The blue stars represent the new STs. PaleVioletRed squares represent the presence of virulence genes and white squares represent the absence of virulence genes, respectively. NA: No allele number assigned.

Based on the *wzi* gene sequences, 239 isolates were classified as 101 different *wzi* allele types (Figure 2). *wzi*150-KL163KL27KL46 (40/239, 16.74%) accounted for the most and *wzi*706, *wzi*727, *wzi*728, *wzi*729, and *wzi*730 were the new *wzi* allele. The *wzi* genotypes of 40 strains were unknown, which might be new alleles. K1 and K2 capsule serotypes commonly found in hvKP were not detected in this study. The results of MLST on these strains showed that 100 different STs were obtained (Figure 2), among which ST2854 (30/239, 12.55%) accounted for the largest proportion, followed by ST5960 (15/239, 6.28%) and ST5915 (13/239, 5.44%) and ST5367, ST5369, ST5370, ST5372, ST5855, ST5874, ST5875, ST5876, ST5914, ST5915, ST5917, ST5918, ST5919, ST5920, ST5947, ST5949, ST5951, ST5952, ST5953, ST5954, ST5955, ST5957, ST5959, ST5960, and ST5961 were the new STs. There are still 30 strains of unknown ST, which might be a new allele. We did not find STs such as ST11 and ST23 that have been reported to have a global epidemic of highly toxic in humans. According to the ST phylogeny tree of 239 isolates (Figure 2), *K. pneumoniae* isolated from five farms in Hubei has a rich variety of serotypes and genotypes. Strains of the same serotype may have differences in the genome and strains of the same genotype may also have different serotypes, such as ST2584 and ST5370 are *wzi*150, isolates of ST5960 have at least 10 different *wzi* types.

### Minimum Spanning Tree Analysis and Prevalence of 100 Sequence Types Among Different Sources and Countries

**FIGURE 3:**
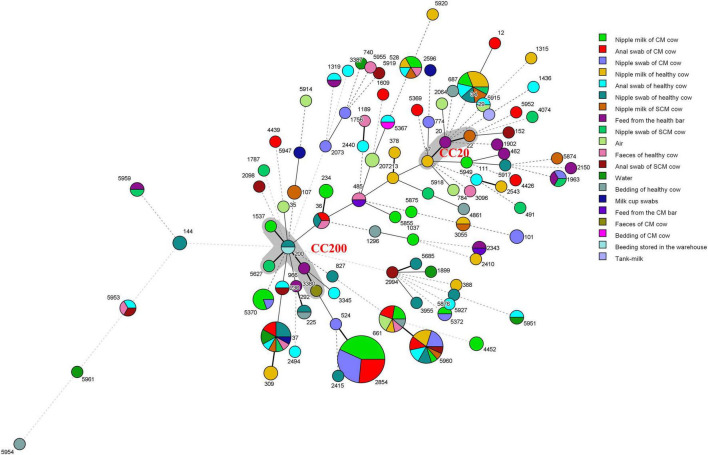
Minimum spanning tree (MST) of 239 *K. pneumoniae*. The gray area represents a clonal complex and the number represents ST. Each ST is grouped by different sample types. One allele difference is represented by a bold line, two allele differences are represented by a straight line, and three or more allele differences are represented by a dotted line.

The MST of 100 different STs showed that there was diversity in the population structure of STs in this study (Figure 3). The founder ST20 and ST17 and ST22 with single locus variants (SLVs) comprise clonal complex 20 (CC20); the founder ST200 and ST292, ST966, ST1537, and ST5627 with SLVs comprise CC200. *K. pneumoniae* isolated from milk, nipple swab, feed, and feces is classified in the same clone complex, which indicated that the contamination of milk may have a great correlation with the hygiene of nipple surface and environment.

**FIGURE 4:**
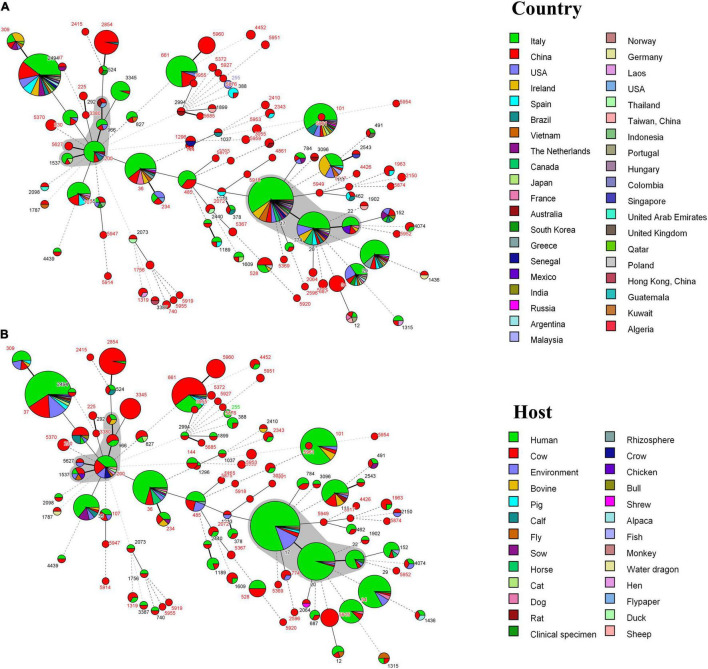
**(A)** MST of 239 *K. pneumoniae* in this study and 722 isolates with the same 104 ST from the PubMLST database. Each ST is grouped by different countries. **(B)** MST of 239 *K. pneumoniae* in this study and 720 isolates with the same 104 ST from the PubMLST database. Each ST is grouped by different hosts. The gray area represents a clonal complex. One allele difference is represented by a bold line, two allele differences are represented by a straight line, and three or more allele differences are represented by a dotted line.

Compared with the PubMLST database, we found that 58 STs were isolated in other countries (Figure 4A). These genotypes are mainly from Italian isolates; ST17, ST20, ST29, ST34, ST36, ST37, and ST101 have a wide range of the region. Furthermore, the host sources of 67 STs were not only cows, but also humans, pigs, chickens, dogs, cats, horses, rhizosphere, environment, and so on. According to Figure 4B, there are 66 STs known to come from multiple hosts, of which 60 STs had existed in humans.

### Virulence Genes

Sixteen of 23 virulence genes were positively detected by PCR (Figure 2). The detection rate of fimbriae synthesis-related gene (*fimH, mrkD*), lipopolysaccharide-related gene (*uge*, *wabG*), iron uptake system (*entB*, *iutA*, and *iroN*), and urease-related gene (*ureA*) was over 85% and others were between 0.42 and 35.56%. Notably, there are 3 isolates [ST34 (*n* = 2), ST2410 (*n* = 1)] carrying *iucA* (Figure 2), which are related to high virulence. Besides, 99.16% (237/239) *K. pneumoniae* carried at least 5 virulence genes and three isolates were positive for 12 virulence genes (Figure 2). There are differences in the virulence genes carried by strains of the same serotype or ST; no significant correlation has been found between the virulence genes and the serotypes or genotypes in this study.

### Antimicrobial Susceptibility Testing

**FIGURE 5:**
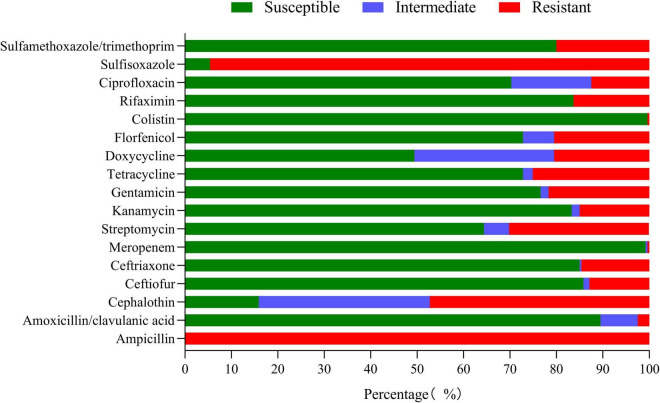
Resistance rate of *K. pneumoniae* to 17 antimicrobials.

**FIGURE 6:**
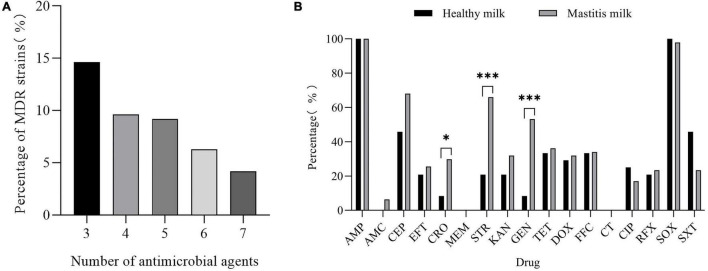
**(A)** Proportion of multidrug-resistant (MDR) *K. pneumoniae* resistant to different amounts of antibiotics. **(B)** Resistance rate of healthy milk and mastitis milk [milk from CM and subclinical mastitis (SCM) cows] to 17 antimicrobials. “*” indicates *p* < 0.05 and “*” indicates *p* < 0.001.

The results of the susceptibility test to 17 antimicrobials are shown in Figure 5 and Supplementary Table 4. *K. pneumoniae* isolates were highly sensitive to meropenem (99.16%) and colistin (99.58%). However, *K. pneumoniae* was completely resistant to ampicillin and highly resistant to sulfisoxazole (94.56%); the resistance rate to cephalothin, streptomycin, gentamicin, tetracycline, doxycycline, florfenicol, and sulfamethoxazole/trimethoprim was between 20.08 and 47.28%. It is worth noting that 43.93% (105/239) of the isolates were MDR strains and neither XDR nor PDR strains were found in this study (Figure 6A). The resistance rate of *K. pneumoniae* to ceftriaxone, streptomycin, and gentamicin in mastitis milk (nipple milk of SCM and CM cows) is significantly higher than that in healthy milk (*p* < 0.05) (Figure 6B). Ninety-three resistance profiles have been identified and the dominant ones were AMP-SOX (57/239, 23.85%), followed by AMP-CEP-SOX (26/239, 10.88%), AMP-STR-GEN-SOX (11/239, 4.66%), and AMP-CEP-STR-GEN-SOX (11/239, 4.66%) (Supplementary Figure 1).

### Antimicrobial Resistance Genes and Quinolone Resistance-Determining Region Mutation

**FIGURE 7:**
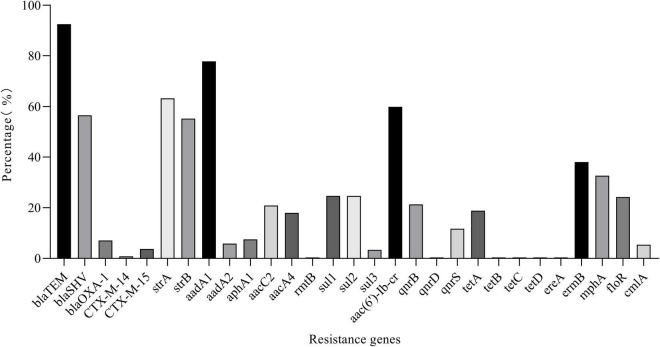
Detection rate of resistance genes.

**FIGURE 8:**
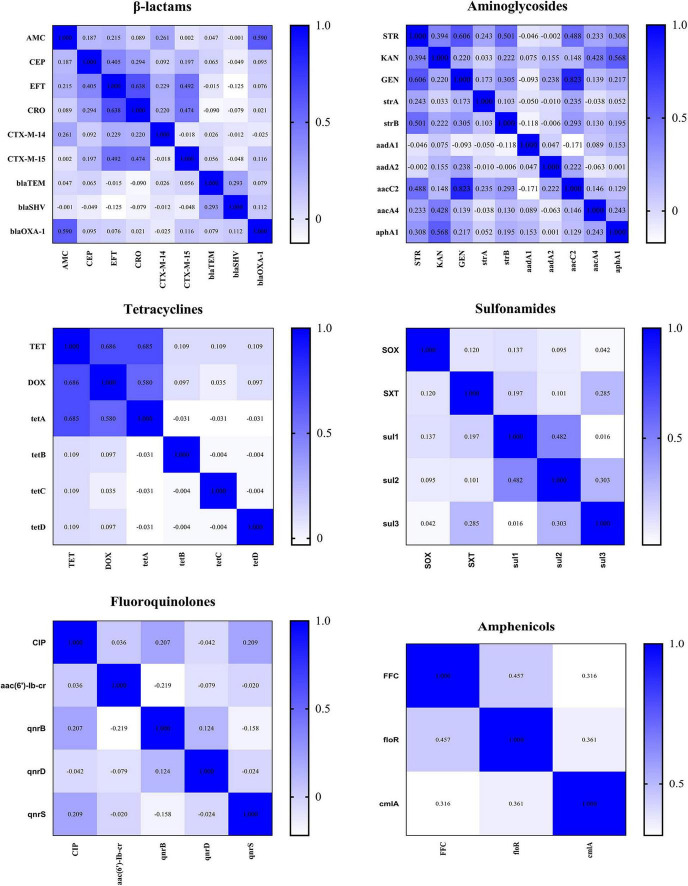
Correlations between different classes of antimicrobials and resistance genes. The intensity of the color indicates the numerical value of the correlation coefficient (r).

Thirty of 68 AMR genes were identified by PCR (Figure 7). The detection rate of *bla*_*TEM*_, *bla*_*SHV*_, *strA*, *strB*, *aadA1*, and *aac(6′)-Ib-cr* was more than 50% and others were between 0.42 and 38.08%. Eleven *CTX-M*-producing *K. pneumoniae* were found. Among the isolates resistant to β-lactam or aminoglycoside antibiotics, more than 90% of isolates have detected at least one β-lactam resistance gene or aminoglycoside resistance gene; among the isolates resistant to TET, DOX, FFC, CIP, and SXT, more than 60% of isolates have detected at least one resistance gene corresponding to the resistance phenotype (Supplementary Figure 1 and Supplementary Table 5). The correlation analysis performed between different phenotypic antibiotic resistance and the antibiotic resistance genes is shown in Figure 8. Among β-lactam antibiotic phenotypes and antibiotic resistance genes, the obtained results revealed low positive correlations between EFT, CRO, and *bla*_*CTX*–M–15_ (*r* = 0.492–0.474) and AMC and *bla*_*OXA–1*_ (*r* = 0.590). Moreover, a low positive correlation was observed between CEP and EFT (*r* = 0.405). Among aminoglycosides, a high positive correlation was found between GEN and *aacC2* (*r* = 0.823); moderate positive correlation was observed between STR and GEN (*r* = 0.606), STR and *strB* (*r* = 0.501), and KAN and *aphA1* (*r* = 0.568); low positive correlation was observed between STR and *aacC2* (*r* = 0.488) and KAN and *aacA4* (*r* = 0.428). Among tetracyclines, moderate positive correlations were observed between TET, DOX, and *tetA* (*r* = 0.580–0.686). Among sulfonamides and amphenicols, low positive correlation was observed between *sul1* and *sul2* (*r* = 0.482), *sul2* and *sul3* (*r* = 0.303), FFC, *floR*, and *cmlA* (*r* = 0.316–0.457). No significant association was found between fluoroquinolone resistance phenotype and PMQR genes. DNA sequencing of QRDR of *gyrA*, *gyrB*, *parC*, and *parE* demonstrated that no amino acid changes were found in these genes whether *K. pneumoniae* isolates were resistant, moderately resistant, or sensitive to ciprofloxacin and silent mutations found are shown in Supplementary Table 6.

## Discussion

*Klebsiella pneumoniae* is an important opportunistic pathogen that has attracted global attention due to the difficult clinical cure of mastitis caused by *K. pneumoniae*, the low effectiveness of antibiotic treatment, and the lack of advancement in preventive measures (Yang et al., 2019b). The prevalence and transmission of multidrug-resistant and hypervirulent *K. pneumoniae* have brought unexpected harm and loss to humans and animals. At present, most of the study on *K. pneumoniae* is in the field of human medicine, while the study data in the field of mastitis is very scarce. This study showed the prevalence and molecular characteristics of virulence, resistance, capsular serotyping, and genotype of *K. pneumoniae* in five cattle farms in Hubei, which will help to track the infection trend of *K. pneumoniae* and emphasized the importance of environmental sanitation in the breeding process. The prevalence of *K. pneumoniae* varied among the five farms involved in this study, which may be related to stocking density and farm environment. The *K. pneumoniae* isolation rate in milk from CM cows was 25.36% (35/138), which was similar to the study by Yang et al. (2021). However, we found that there was no significant difference in the *K. pneumoniae* prevalence of nipple milk, skin swabs, and anal swabs between healthy and mastitis cows, which was different from the previous study (Koovapra et al., 2016). This may be due to that most of the CM cows are in the period of treatment or recovery. The results also confirmed that *K. pneumoniae*, as an environmental pathogen, is also ubiquitous in the environment (Wareth and Neubauer, 2021).

At present, the hvKP was reported to be most common in *K. pneumoniae* with ST11, serotype K1 or K2 (Wang et al., 2021). No strains of these serotypes and sequence types were found in this study, but K54 (*wzi*66, *wzi*115, *n* = 2) and K57 (*wzi*57, *n* = 1) were found, which may also be related to high virulence (Zhong et al., 2020). The genetic diversity of *K. pneumoniae* is very rich and its serotypes and sequence types are not in a one-to-one correspondence, which is similar to previous studies (Benulič et al., 2020; Lepuschitz et al., 2020). Many studies confirmed that the occurrence of *K. pneumoniae* is related to the environment of farms and *K. pneumoniae* can be spread through contaminated feed, feces, drinking water, and so on (Wareth and Neubauer, 2021; Zhao et al., 2021). Similar results were also observed in our MST analysis. *K. pneumoniae* isolated from milk, nipple swab, feed, and feces is closely related and 58 STs exist in different countries and 66 STs have been found in different hosts. Strains of these genotypes have potential harm of cross-species and regional transmission, which cannot be ignored. Enhancing the sanitation of the breeding environment and disinfection of the cattle stalls may be an effective ways to prevent mastitis.

The pathogenicity of *K. pneumoniae* is inseparable from the role of virulence factors. In this study, *peg-344*, *iroB*, *_*p*_rmpA*, and *_*p*_rmpA2* related to high virulence were not found, but 3 strains were detected carrying the *iucA*; further study is needed to verify the virulence, such as animal experiments. In addition, PCR detection results showed that fimbriae-related genes (*fimH*, *mrkD*), iron uptake system (*iutA*, *iroN*, and *entB*), urease-related genes (*ureA*), and the lipopolysaccharide-related genes (*uge*, *wabG*) were widely distributed in the isolates, which were similar to the previous results (Zhang et al., 2018; Yang et al., 2019a). These results can preliminarily elucidate the pathogenic mechanism of the *K. pneumoniae* that may include: synthesizing fimbriae to adhere to the surface of host cells or form biofilms for virulence, secreting siderophores to absorb iron in the host for metabolism to enhance virulence, evading serum killing of phagocytes and suppress host immunity by utilizing capsular polysaccharides, and lipopolysaccharide aggregates into complexes on the surface of the *K. pneumoniae* so that the bacteria can escape or resist the killing of the host’s innate immunity (Schroll et al., 2010; Li et al., 2014; Liu et al., 2019).

Antibiotic resistance has always been a key and difficult issue of global concern. The results of antibiotic susceptibility test showed that *K. pneumoniae* isolates were highly sensitive to meropenem and colistin. It is mainly because carbapenems are strictly forbidden to be used in animals; the Chinese government has officially banned polymyxin as an animal growth promoter on 30 April 2017 (Wang Y. et al., 2020; Yang et al., 2021). However, *K. pneumoniae* has different degrees of resistance to other antibiotics and has a complex antimicrobial spectrum. As previously reported, *K. pneumoniae* shows intrinsic resistance to ampicillin (Fu et al., 2007). The rate of MDR (43.93%) and resistance to SOX (94.56%) are higher than that of the study by Zhang et al. (2020). β-lactam and aminoglycoside antibiotics have long been used to treat mastitis in five farms, which may explain why the resistance rate of isolates in mastitis milk to CEP, STR, and GEN was significantly higher than that of healthy milk. The development of antibiotic resistance is inseparable from the existence and spread of resistance genes. Our results show that *bla*_*TEM*_ and *bla*_*SHV*_ are present in mostly *K. pneumoniae* and *bla*_*CTX–M–15*_ is the main *CTX-M* gene detected, which is similar to the results of previous studies (Timofte et al., 2014; Carvalho et al., 2021). These extended-spectrum β-lactamase (ESBL) resistance genes can lead to resistance by hydrolysis of penicillins and cephalosporins (Algammal et al., 2020a). Correlation analysis showed that the resistance of *K. pneumoniae* to AMC, EFT, and CRO may be caused by hydrolysis mediated by *bla*_*OXA–1*_ and *bla*_*CTX–M–15*_. A variety of aminoglycoside-modifying enzyme genes (*aadA1*, *aacc2*, *aacA4*, and *aphA1*) were detected in the isolates. These enzymes can modify the active antimicrobial drugs that enter the cell and make them inactive (Belaynehe et al., 2017). The resistance of STR is mainly mediated by *strA* and *strB* and correlation analysis shows that the resistance of the STR of *K. pneumoniae* in this study may be more related to *strB.* It has been reported that *tetA* and *floR*/*cmlA* mediate the efflux of tetracycline and florfenicol, respectively, which can pump the drug out of the cell and reduce the intracellular drug concentration to generate resistance (Acosta-Pérez et al., 2015; Wang et al., 2018; Graesbøll et al., 2019). Notably, *tetA* and *floR* are the most frequently observed AMR genes in *K. pneumoniae* resistant to tetracycline and amide alcohols, respectively, which are consistent with the study by Nobrega et al. (2021). Although the three dihydrofolate synthase genes (*sul1*, *sul2*, and *sul3*) were detected to varying degrees, no significant correlation was found between them and SOX and SXT. Other resistance mechanisms, such as the expression of dihydrofolate reductase gene (*dfr*) and permeability barriers, are the causes of sulfonamide resistance (Skold, 2001). *K. pneumoniae* can be against quinolones through several mechanisms, including mutations in quinolone resistance-determining regions, plasmid-mediated quinolone resistance (PMQR), increased activity of efflux pumps, and decreased cellular permeability. Among them, mutation of QRDR is the main mechanism mediating quinolone resistance, especially *gyrA* and *parC* (Kareem et al., 2021). However, no amino acid changes were found in this study, which is different from the study by Saiful Anuar et al. (2013) and Fu et al. (2008), but similar to the study by Kim et al. (2018). Although the PMQR gene was detected to varying degrees in the isolates, its correlation with the ciprofloxacin resistance phenotype was not significant. We speculate that the activity of efflux pumps and the reduction of cell permeability may be the main reasons for mediating *K. pneumoniae* resistance to ciprofloxacin, but further verification is needed.

## Conclusion

The occurrence of mastitis may be closely related to environmental hygiene. Virulence factors such as fimbriae, iron uptake, and lipopolysaccharide may play important roles in the pathogenesis of *K. pneumoniae*. The MDR of *K. pneumoniae* is a serious public health problem that still needs to be paid attention to, especially, the high resistance caused by the frequent use of β-lactams, aminoglycosides, and sulfonamides. The resistance of some antibiotics is attributed to the existence of resistance genes and other mechanisms such as efflux pumps and decreased permeability may be involved in the resistance of *K. pneumoniae* to sulfonamides and fluoroquinolones. Multiple sequence types of *K. pneumoniae* have the risk of cross-species and regional transmission. It is recommended to strengthen and regularly conduct surveillance, antibiotic resistance investigation, and traceability study on this strain. Further studies will be required to clarify whether the resistance and virulence characteristics of these isolates are affected by some movable genetic elements (such as plasmids) and whether they pose a risk of transmission.

## Data Availability Statement

The original contributions presented in the study are included in the article/Supplementary Material, further inquiries can be directed to the corresponding author/s.

## Author Contributions

YW conceived the study and directed the study. XW, JL, JF, YF, RG, SH, and MZ performed the experiments. GW performed the somatic cell count of milk. XW performed the data analysis and wrote the manuscript. YW, HH, GC, and MS revised the manuscript. All authors have read and approved the final version of the manuscript.

## Conflict of Interest

The authors declare that the research was conducted in the absence of any commercial or financial relationships that could be construed as a potential conflict of interest.

## Publisher’s Note

All claims expressed in this article are solely those of the authors and do not necessarily represent those of their affiliated organizations, or those of the publisher, the editors and the reviewers. Any product that may be evaluated in this article, or claim that may be made by its manufacturer, is not guaranteed or endorsed by the publisher.

## Footnotes

1
http://www.pasteur.fr/mlst

2
https://bigsdb.pasteur.fr/cgi-bin/bigsdb/bigsdb.pl?db=pubmlst_klebsiella_seqdef&page=sequenceQuery

3
http://bigsdb.pasteur.fr/perl/bigsdb/bigsdb.pl?db=pubmlst_klebsiella_seqdef_public&page=profiles

4
https://itol.embl.de
