# Supplementary material for: Epidemiology, Environmental Risks, Virulence, and Resistance Determinants of Klebsiella pneumoniae From Dairy Cows in Hubei, China
Source: Front Microbiol. 2022 May 4;13:858799. doi: 10.3389/fmicb.2022.858799 (PMC9117759; doi:10.3389/fmicb.2022.858799)
Supplement: Supplementary file 1 [file Data_Sheet_1.docx]

Supplementary Material

# Supplementary Data

**Figure S1** **(A)**The resistance phenotypes and resistance genes of 239 *K. pneumoniae* of β-lactams. **(B)**The resistance phenotypes and resistance genes of 239 *K. pneumoniae* of aminoglycosides. **(C)**The resistance phenotypes and resistance genes of 239 *K. pneumoniae* of tetracyclines. **(D)**The resistance phenotypes and resistance genes of 239 *K. pneumoniae* of quinolones. **(E)**The resistance phenotypes and resistance genes of 239 *K. pneumoniae* of sulfonamides. **(F)**The resistance phenotypes and resistance genes of 239 *K. pneumoniae* of phenicols. **(G)**The resistance phenotypes and resistance genes of 239 *K. pneumoniae* of polymyxins and rifamycins . The magenta filled square represent resistance to the antibiotic and brown filled circle represent the presence of resistance genes, respectively white unfilled square and circle represents non-resistant and absence.

**Table S1** Primers of virulence genes in this study

**Table S2** Primers of AMR genes in this study

**Table S3** The prevalence of *K. pneumoniae* in different sample types in five farms

**Table S4** MIC distribution of 239 *K. pneumoniae* to 17 antimicrobials

**Table S5** The frequency of the phenotypic multidrug-resistance and the antibiotic-resistance genes among the retrieved strains (n = 105)

**Table S6** Silent mutations of *gyrA*, *gyrB*, *parC*, *parE* in 30 ciprofloxacin-resistant, 3 ciprofloxacin-intermediate and 7 ciprofloxacin-sensitive *K. pneumoniae*

**A** *
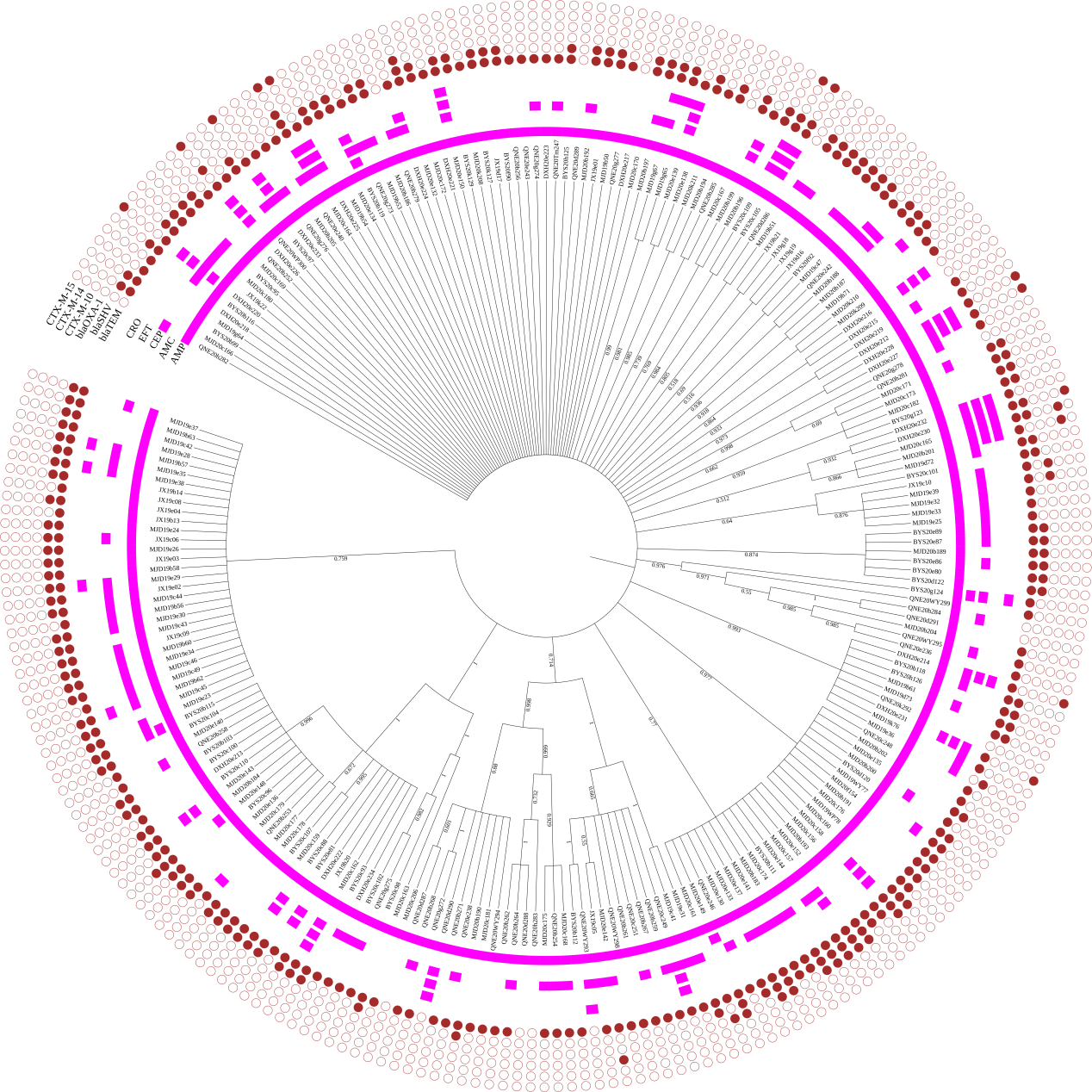
*

**B**

**
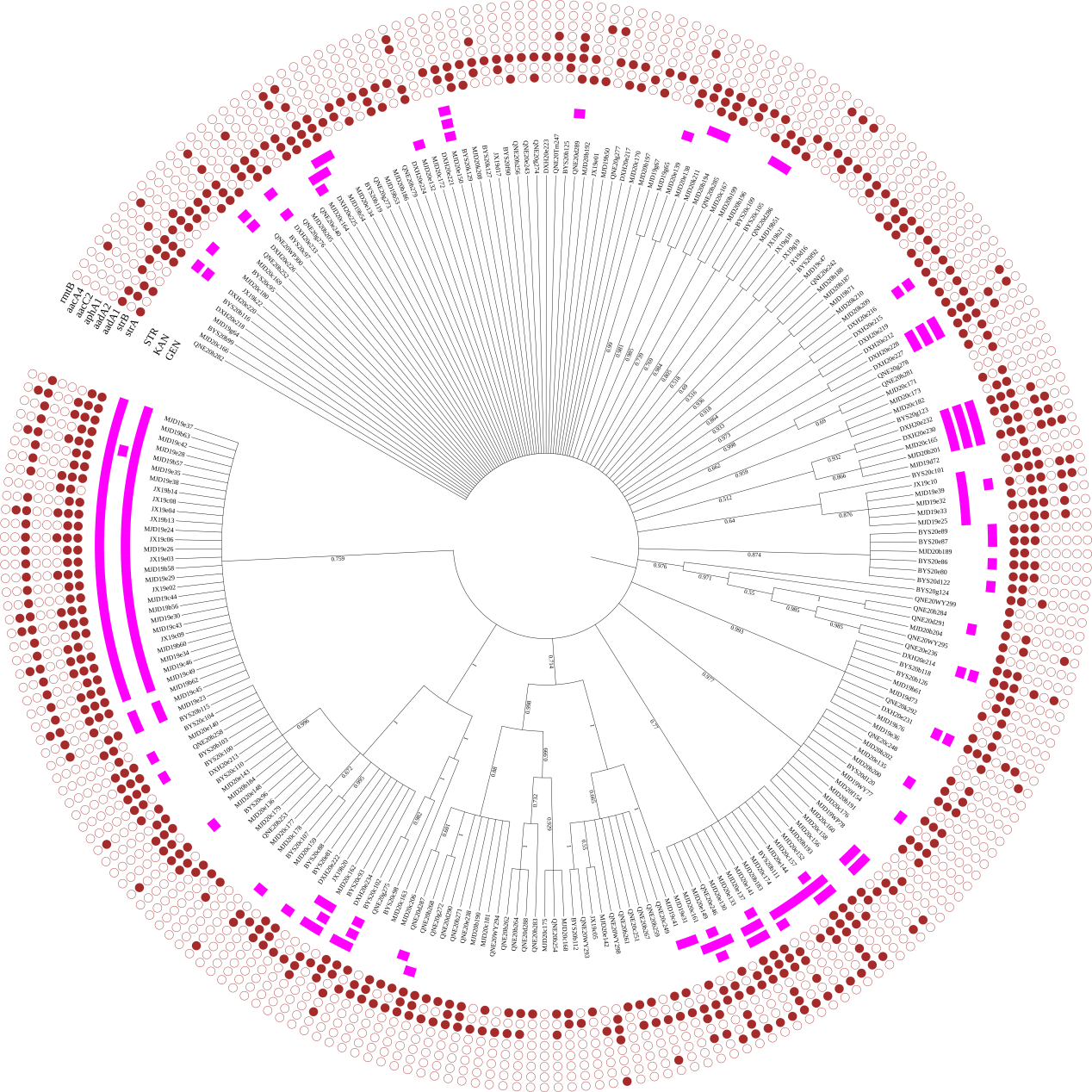
**

**C** *
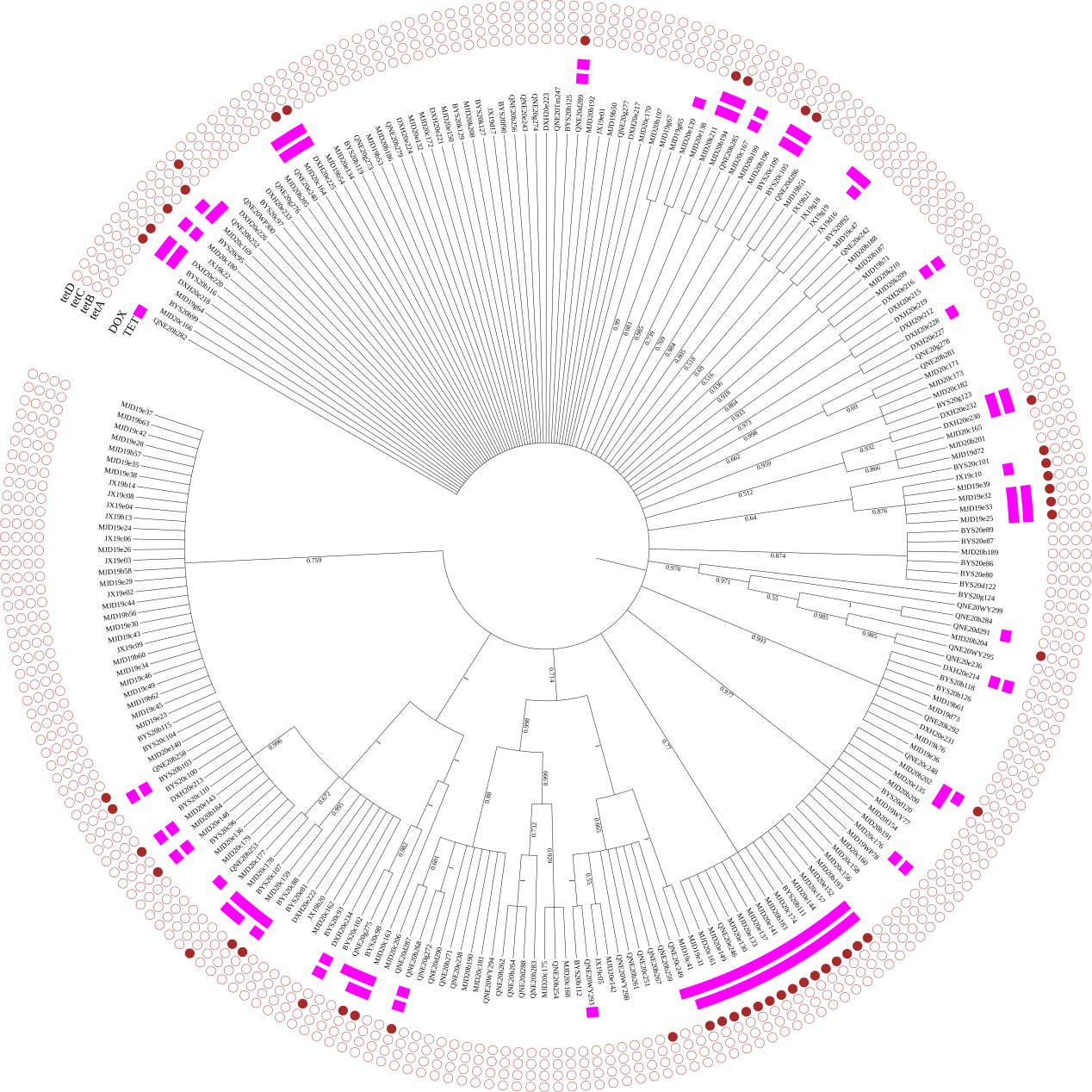
*

**D**

**
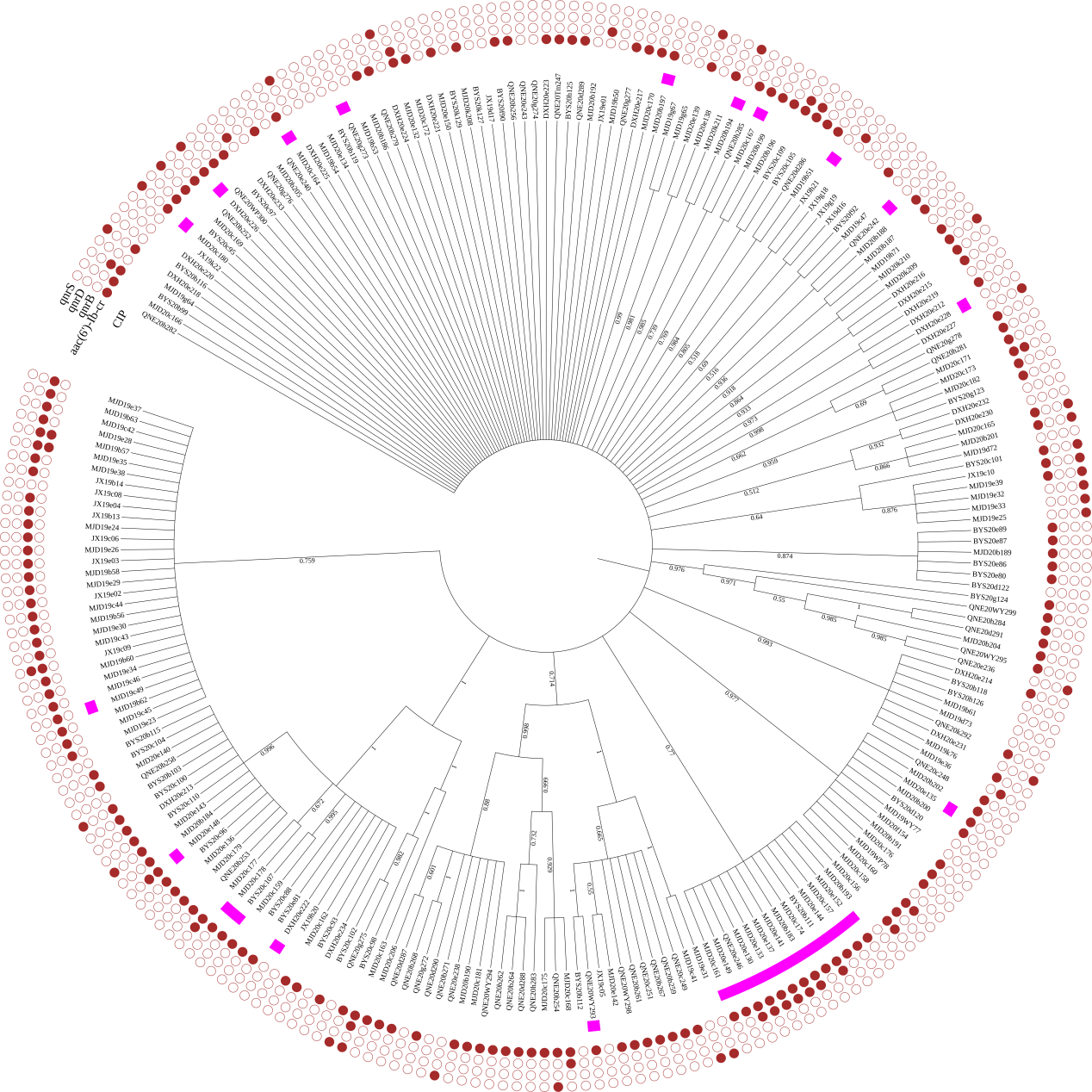
**

**E**

**
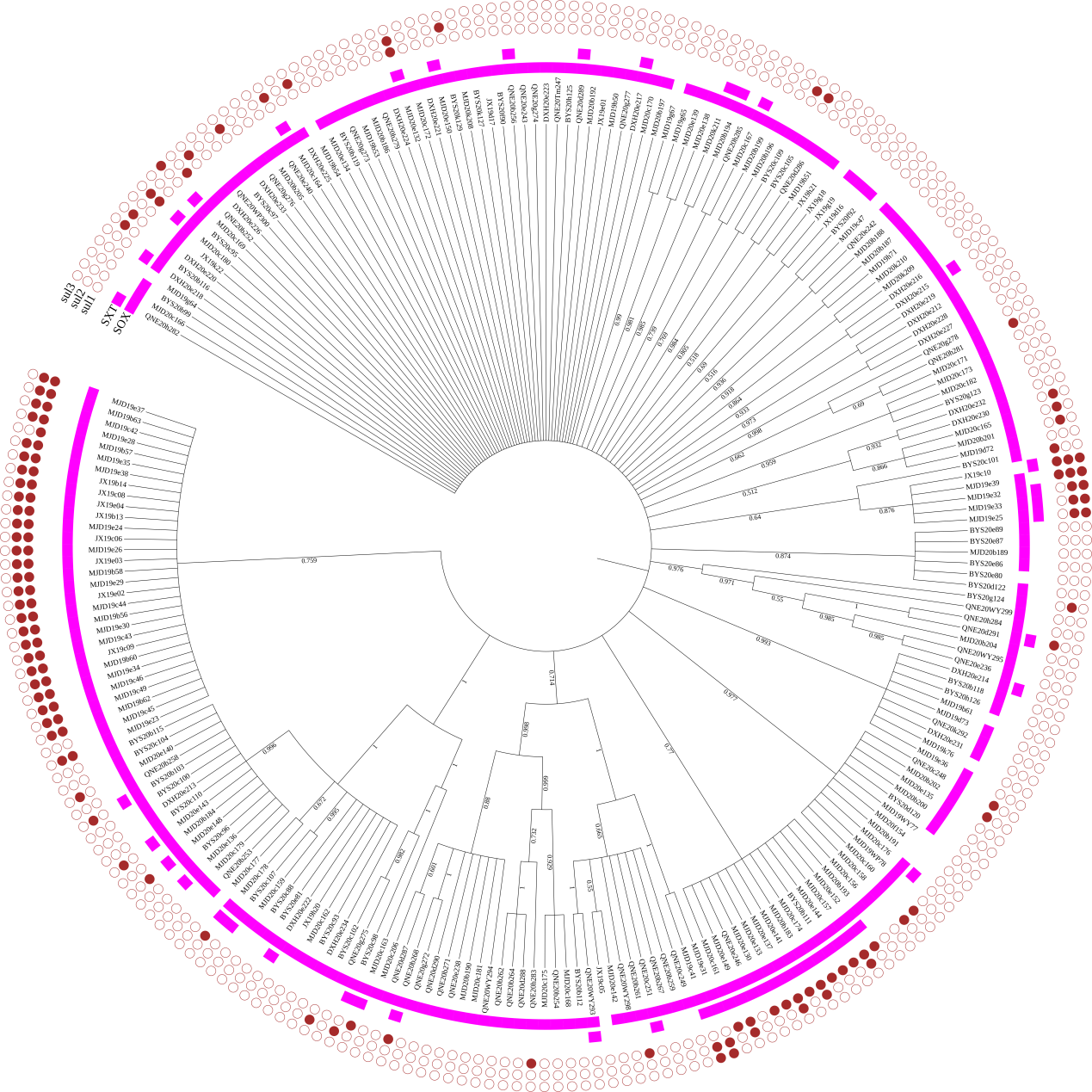
**

**F**

**
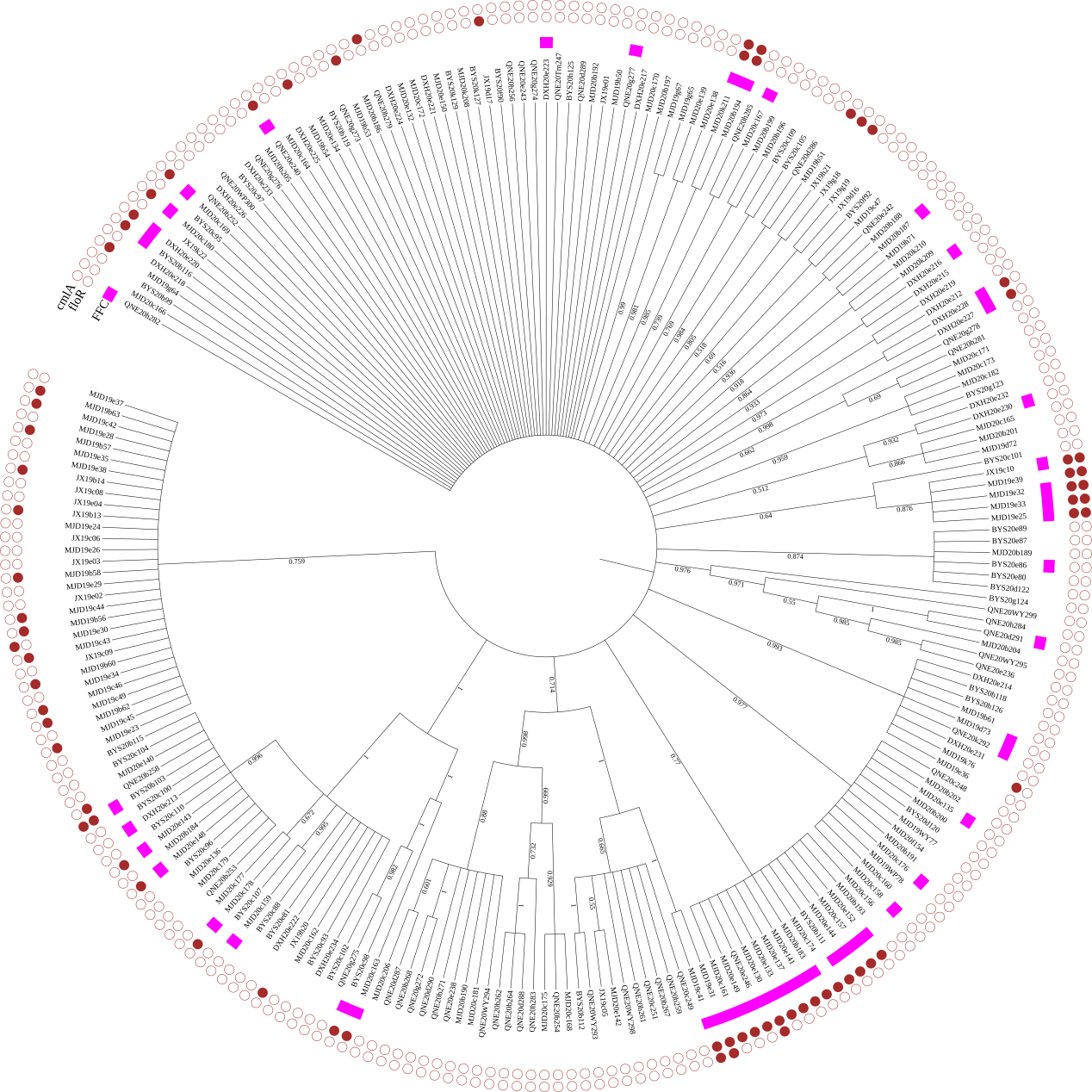
**

**G**

**
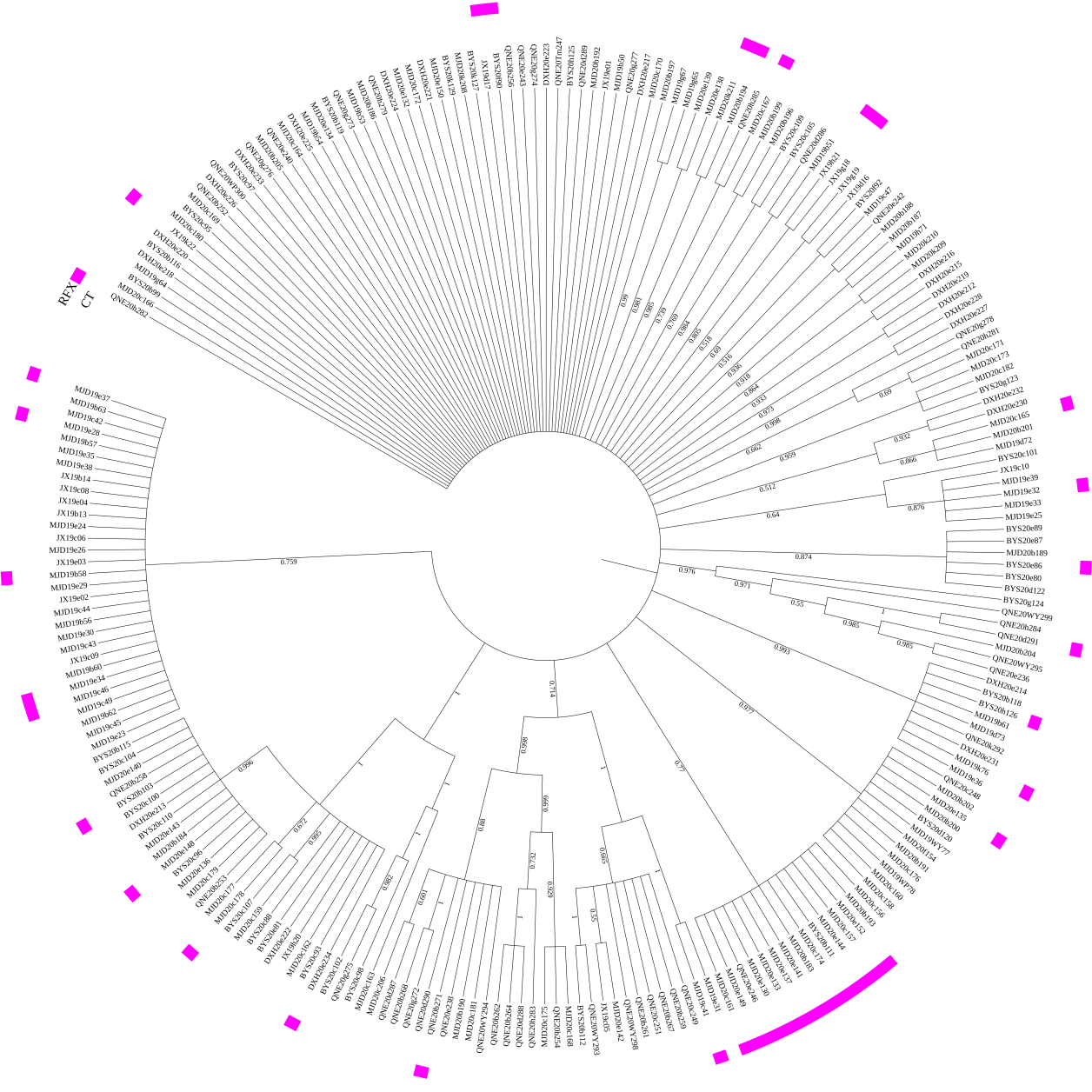
**

**Figure S1** **(A)**The resistance phenotypes and resistance genes of 239 *K. pneumoniae* of β-lactams. **(B)**The resistance phenotypes and resistance genes of 239 *K. pneumoniae* of aminoglycosides. **(C)**The resistance phenotypes and resistance genes of 239 *K. pneumoniae* of tetracyclines. **(D)**The resistance phenotypes and resistance genes of 239 *K. pneumoniae* of quinolones. **(E)**The resistance phenotypes and resistance genes of 239 *K. pneumoniae* of sulfonamides. **(F)**The resistance phenotypes and resistance genes of 239 *K. pneumoniae* of phenicols. **(G)**The resistance phenotypes and resistance genes of 239 *K. pneumoniae* of polymyxins and rifamycins . The magenta filled square represent resistance to the antibiotic and brown filled circle represent the presence of resistance genes, respectively white unfilled square and circle represents non-resistant and absence.

**Table S1** Primers of virulence genes in this study

| Gene type | Primes^a^ | | Sequence (5’-3’) | AL^b^ (bp) | AT^c^ (℃) | Reference |
| --- | --- | --- | --- | --- | --- | --- |
| Transporter | *peg344* | F | AAAGGACAGAAAGCCAGTG | 411 | 53 | (Harada and Doia, 2018) |
|  |  | R | CAATGACGAGGGGGATAATC |  |  |  |
| Fimbriae synthesis- related | *fimH* | F | TGCTGCTGGGCTGGTCGATG | 688 | 60 | (Xu SB, Yang SY, Weng SS, Chen C, Zhang WH, 2019) |
|  |  | R | GGGAGGGTGACGGTGACATC |  |  |  |
|  | *mrkD* | F | AAGCTATCGCTGTACTTCCGGCA | 340 | 60 | (Lan et al., 2019) |
|  |  | R | GGCGTTGGCGCTCAGATAGG |  |  |  |
| Lipopolysaccharide-related | *uge* | F | TCTTCACGCCTTCCTTCACT | 534 | 53 | (Osman et al., 2014) |
|  |  | R | GATCATCCGGTCTCCCTGTA |  |  |  |
|  | *wabG* | F | ACCATCGGCCATTTGATAGA | 683 | 53 | (Xu SB, Yang SY, Weng SS, Chen C, Zhang WH, 2019) |
|  |  | R | CGGACTGGCAGATCCATATC |  |  |  |
| Capsular polysaccharide synthesis and synthesis regulation related | *wcaG* | F | GGTTGGGTCAGCAATCGTA | 169 | 58 | (Derakhshan et al., 2016) |
|  |  | R | ACTATTCCGCCAACTTTTGC |  |  |  |
|  | *_c_rmpA* | F | GTAATAGAGATATAAATATCATATTGA | 588 | 50 | (Harada and Doia, 2018) |
|  |  | R | CATCTTTCATCAACCATTTC |  |  |  |
|  | *_p_rmpA* | F | GAGTAGTTAATAAATCAATAGCAAT | 332 | 50 | (Harada and Doia, 2018) |
|  |  | R | CAGTAGGCATTGCAGCA |  |  |  |
|  | *_p_rmpA2* | F | GTGCAATAAGGATGTTACATTA | 430 | 50 | (Harada and Doia, 2018) |
|  |  | R | GGATGCCCTCCTCCTG |  |  |  |
|  | *magA* | F | GGTGCTCTTTACATCATTGC | 1280 | 55 | (Osman et al., 2014) |
|  |  | R | GCAATGGCCATTTGCGTTAG |  |  |  |
| Iron uptake system | *iroB* | F | ATCTCATCATCTACCCTCCGCTC | 235 | 59 | (Harada and Doia, 2018) |
|  |  | R | GGTTCGCCGTCGTTTTCAA |  |  |  |
|  | *iroN* | F | GGAGCTCGGCGATATGTTCA | 369 | 63 | This study |
|  |  | R | AGCAGATTTCCTTCCAGCCC |  |  |  |
|  | *aerobactin* | F | GCATAGGCGGATACGAACAT | 556 | 55 | (Osman et al., 2014) |
|  |  | R | CACAGGGCAATTGCTTACCT |  |  |  |
|  | *iutA* | F | CCTTCTATCCGTTCCCGACG | 352 | 63 | This study |
|  |  | R | CGGTTTTCGGTCCACTGGTA |  |  |  |
|  | *irp2* | F | GCTACAATGGGACAGCAACGAC | 230 | 59 | (Harada and Doia, 2018) |
|  |  | R | GCAGAGCGATACGGAAAATGC |  |  |  |
|  | *iucA* | F | GCTTATTTCTCCCCAACCC | 583 | 59 | (Harada and Doia, 2018) |
|  |  | R | TCAGCCCTTTAGCGACAAG |  |  |  |
|  | *ybtA* | F | ATGACGGAGTCACCGCAAAC | 960 | 55 | (Xu SB, Yang SY, Weng SS, Chen C, Zhang WH, 2019) |
|  |  | R | TTACATCACGCGTTTAAAGG |  |  |  |
|  | *kfu* | F | GAAGTGACGCTGTTTCTGGC | 797 | 55 | (Osman et al., 2014) |
|  |  | R | TTTCGTGTGGCCAGTGACTC |  |  |  |
|  | *entB* | F | ATTTCCTCAACTTCTGGGGC | 371 | 60 | (Candan and Aksöz, 2015) |
|  |  | R | AGCATCGGTGGCGGTGGTCA |  |  |  |
| Urease-related | *allS* | F | CCGAAACATTACGCACCTTT | 508 | 49 | (Xu SB, Yang SY, Weng SS, Chen C, Zhang WH, 2019) |
|  |  | R | ATCACGAAGAGCCAGGTCAC |  |  |  |
|  | *ureA* | F | GCTGACTTAAGAGAACGTTATG | 337 | 55 | (He, 2012) |
|  |  | R | GATCATGGCGCTACCT(C/T)A |  |  |  |
| Tellurite resistance gene | *terB* | F | TATCGCTGTTGCCAGTGAC | 235 | 53 | (Harada and Doia, 2018) |
|  |  | R | AATACCGACACGAAGAGCC |  |  |  |
| Hemolysin | *hly* | F | AACAAGGATAAGCACTGTTCTGGCT | 1177 | 58 | (Candan and Aksöz, 2015) |
|  |  | R | ACCATATAAGCGGTCATTCCCGTCA |  |  |  |

^a^ Forward, F; Reverse, R.

^b^ Amplicon length, AL.

^c^ Annealing temperature, AT.

**Table S2** Primers of AMR genes in this study

| Gene type | Primes^a^ | | Sequence (5’-3’) | AL^b^ (bp) | AT^c^ (℃) | Refence |
| --- | --- | --- | --- | --- | --- | --- |
| ESBLs | *bla*_CTX-M-2_ | F | AAATGTGCTGCTCCTTTCGTGAGC | 1122 | 55 | (Andrade et al., 2018) |
|  |  | R | AGGGTTCGTTGCAAGACAAGACTG |  |  |  |
|  | *bla*_CTX-M-10_ | F | CCGCGCTACACTTTGTGGC | 944 | 55 | (Kuang, 2015) |
|  |  | R | TTACAAACCGTTGGTGACG |  |  |  |
|  | *bla*_CTX-M-14_ | F | TACCGCAGATAATACGCAGGTG | 355 | 55 | (Huang et al., 2012) |
|  |  | R | CAGCGTAGGTTCAGTGCGATCC |  |  |  |
|  | *bla*_CTX-M-15_ | F | GACGATGTCACTGGCTGAGC | 499 | 55 | (Andrade et al., 2018) |
|  |  | R | AGCCGCCGACGCTAATACA |  |  |  |
|  | *bla*_SHV_ | F | ATGCGTTATATTCGCCTGTG | 747 | 55 | (Zhang et al., 2016) |
|  |  | R | TGCTTTGTTATTCGGGCCAA |  |  |  |
|  | *bla*_TEM_ | F | TCGCCGCATACACTATTCTCAGAATGA | 445 | 55 | (Zhang et al., 2016) |
|  |  | R | ACGCTCACCGGCTCCAGATTTAT |  |  |  |
|  | *bla*_OXA-1_ | F | ACACAATACATATCAACTTCGC | 813 | 55 | (Riaño et al., 2006) |
|  |  | R | AGTGTGTTTAGAATGGTGATC |  |  |  |
| Carbapenem resistance gene | *IMP* | F | GGAATAGAGTGGCTTAAYTCTC | 232 | 55 | (Candan and Aksöz, 2015) |
|  |  | R | GGTTTAAYAAAACAACCACC |  |  |  |
|  | *VIM* | F | GATGGTGTTTGGTCGCATA | 390 | 55 | (Candan and Aksöz, 2015) |
|  |  | R | CGAATGCGCAGCACCAG |  |  |  |
|  | *OXA-48* | F | GCGTGGTTAAGGATGAACAC | 438 | 55 | (Candan and Aksöz, 2015) |
|  |  | R | CATCAAGTTCAACCCAACCG |  |  |  |
|  | *OXA-181* | F | TCAGTAGCTGAACAGGAGGGA | 1461 | 55 | (Liu et al., 2020) |
|  |  | R | TTCGAGCCGCAGACAATT |  |  |  |
|  | *NDM* | F | GGTTTGGCGATCTGGTTTTC | 621 | 55 | (Poirel et al., 2011) |
|  |  | R | CGGAATGGCTCATCACGATC |  |  |  |
|  | *KPC* | F | CGTCTAGTTCTGCTGTCTTG | 798 | 55 | (Candan and Aksöz, 2015) |
|  |  | R | CTTGTCATCCTTGTTAGGCG |  |  |  |
| AmpC | *MOX* | F | GCTGCTCAAGGAGCACAGGAT | 520 | 55 | (Chuong et al., 2018) |
|  |  | R | CACATTGACATAGGTGTGGTGC |  |  |  |
|  | *CIT* | F | CGAAGAGGCAATGACCAGAC | 538 | 55 | (Fadare et al., 2020) |
|  |  | R | ACGGACAGGGTTAGGATAGY |  |  |  |
|  | *DHA* | F | GCCTGTTTGGTGCTCTGA | 460 | 55 | (Gharout-Sait et al., 2014) |
|  |  | R | GCACGGTTATACGGCTGA |  |  |  |
|  | *ACC* | F | AACAGCCTCAGCAGCCGGTTA | 346 | 55 | (Chuong et al., 2018) |
|  |  | R | TTCGCCGCAATCATCCCTAGC |  |  |  |
|  | *EBC* | F | CGGTAAAGCCGATGTTGCG | 683 | 55 | (Fadare et al., 2020) |
|  |  | R | AGCCTAACCCCTGATACA |  |  |  |
|  | *FOX* | F | AACATGGGGTATCAGGGAGATG | 190 | 55 | (Chuong et al., 2018) |
|  |  | R | CAAAGCGCGTAACCGGATTGG |  |  |  |
| Aminoglycoside resistance gene | *armA* | F | CAAATGGATAAGAATGATGTT | 774 | 55 | (Granier et al., 2011) |
|  |  | R | TTATTTCTGAAATCCACT |  |  |  |
|  | *rmtA* | F | ATGAGCTTTGACGATGCCCTA | 756 | 55 | (Granier et al., 2011) |
|  |  | R | TCACTTATTCCTTTTTATCATG |  |  |  |
|  | *rmtB* | F | ATGAACATCAACGATGCCCT | 769 | 55 | (Granier et al., 2011) |
|  |  | R | CCTTCTGATTGGCTTATCCA |  |  |  |
|  | *rmtC* | F | CGAAGAAGTAACAGCCAAAG | 711 | 55 | (Granier et al., 2011) |
|  |  | R | ATCCCAACATCTCTCCCACT |  |  |  |
|  | *rmtD* | F | CGGCACGCGATTGGGAAGC | 401 | 55 | (Granier et al., 2011) |
|  |  | R | CGGAAACGATGCGACGAT |  |  |  |
|  | *rmtE* | F | ATGAATATTGATGAAATGGTTGC | 818 | 55 | (Granier et al., 2011) |
|  |  | R | TGATTGATTTCCTCCGTTTTTG |  |  |  |
|  | *npmA* | F | CTCAAAGGAACAAAGACGG | 641 | 55 | (Kuang, 2015) |
|  |  | R | GAAACATGGCCAGAAACTC |  |  |  |
|  | *aadA1* | F | TATCCAGCTAAGCGCGAAC | 447 | 55 | (Tabaran et al., 2017) |
|  |  | R | ATTTGCCGACTACCTTGGT |  |  |  |
|  | *aadA2* | F | CATCCCGTGGCGTTATCC | 370 | 55 | (Ma, 2014) |
|  |  | R | CTGGGCAGGTAGGCGTTT |  |  |  |
|  | *aadB* | F | ATGGACACAACGCAGGTCGC | 534 | 55 | (Al-Marzooq et al., 2015) |
|  |  | R | TTAGGCCGCATATCGCGACC |  |  |  |
|  | *aacC1* | F | ATGGGCATCATTCGCACATGTAGG | 861 | 55 | (Al-Marzooq et al., 2015) |
|  |  | R | TTAGGTGGCGGTACTTGGGTC |  |  |  |
|  | *aacC2* | F | ATGCATACGCGGAAGGCAATAAC | 861 | 55 | (Al-Marzooq et al., 2015) |
|  |  | R | CTAACCGGAAGGCTCGCAAG |  |  |  |
|  | *aac(3)-Ⅳ* | F | TCGATGGGCAGGTACTTCTC | 230 | 55 | (Almaghrabi et al., 2014) |
|  |  | R | ACCGACTGGACCTTCCTTCT |  |  |  |
|  | *aacA4* | F | TATGAGTGGCTAAATCGAT | 395 | 55 | (Huang et al., 2012) |
|  |  | R | CCCGCTTTCTCGTAGCA |  |  |  |
|  | *aphA1* | F | ATGGGCTCGCGATAATGTC | 600 | 55 | (Sáenz et al., 2004) |
|  |  | R | CTCACCGAGGCAGTTCCAT |  |  |  |
|  | *aphA2* | F | GAACAAGATGGATTGCACGC | 680 | 55 | (Sáenz et al., 2004) |
|  |  | R | GCTCTTCAGCAATATCACGG |  |  |  |
|  | *aphA6* | F | ATGGAATTGCCCAATATTATTC | 780 | 55 | (Al-Marzooq et al., 2015) |
|  |  | R | TCAATTCAATTCATCAAGTTTTA |  |  |  |
|  | *strA* | F | CTTGGTGATAACGGCAATTC | 548 | 55 | (Fazel et al., 2019) |
|  |  | R | CCAATCGCAGATAGAAGGC |  |  |  |
|  | *strB* | F | ATCGTCAAGGGATTGAAACC | 509 | 55 | (Fazel et al., 2019) |
|  |  | R | GGATCGTAGAACATATTGGC |  |  |  |
| Quinolone resistance gene | *aac(6′)-Ib-cr* | F | TTGCGATGCTCTATGAGTGGCTA | 482 | 55 | (Park et al., 2006) |
|  |  | R | CTCGAATGCCTGGCGTGTTT |  |  |  |
|  | *qnrA* | F | TCAGCAAGCGGATTTCTCA | 608 | 55 | (Yang et al., 2019) |
|  |  | R | GGCAGCACTATTACTCCCA |  |  |  |
|  | *qnrB* | F | AGTCGTGCGATGCTGAAAGA | 333 | 55 | This study |
|  |  | R | TCGCCAGTCGAAAGTCGAAA |  |  |  |
|  | *qnrC* | F | TGAATTATTCCCATAAAACG | 654 | 55 | (Veldman et al., 2011) |
|  |  | R | TGTCTGGAATAACAATCACC |  |  |  |
|  | *qnrD* | F | CGGGGAATAGAGTTAAAAAT | 598 | 55 | (Veldman et al., 2011) |
|  |  | R | TATCGGTGAACAATAACACC |  |  |  |
|  | *qnrS* | F | CGACGTGCTAACTTGCGTGATA | 538 | 55 | (Kuang, 2015) |
|  |  | R | TACCCAGTGCTTCGAGAATCAG |  |  |  |
| Tetracycline resistance gene | *tetA* | F | ATTGCTCGGGCGAGTGAATG | 794 | 55 | This study |
|  |  | R | TGAAACCCAACAGACCCCTG |  |  |  |
|  | *tetB* | F | CCTCAGCTTCTCAACGCGTG | 634 | 55 | (Tabaran et al., 2017) |
|  |  | R | GCACCTTGCTGATGACTCTT |  |  |  |
|  | *tetC* | F | ACTTGGAGCCACTATCGAC | 881 | 55 | (Maynard et al., 2004) |
|  |  | R | CTACAATCCATGCCAACCC |  |  |  |
|  | *tetD* | F | CAGGCATTCAGTATTGCCGC | 227 | 55 | This study |
|  |  | R | TGGATGTTGTATCTCGGGCG |  |  |  |
|  | *tetE* | F | GTTATTACGGGAGTTTGTTGG | 278 | 55 | (Li et al., 2016) |
|  |  | R | AATACAACACCCACACTACGC |  |  |  |
|  | *tetG* | F | TGCTTGGACAGCTTTCGGAT | 411 | 55 | This study |
|  |  | R | GTTTGATGCGAACCGGCTTT |  |  |  |
| Sulfonamide resistance gene | *Sul1* | F | GTGACGGTGTTCGGCATTCT | 779 | 55 | (Fazel et al., 2019) |
|  |  | R | TCCGAGAAGGTGATTGCGCT |  |  |  |
|  | *Sul2* | F | CGGCATCGTCAACATAACCT | 721 | 55 | (Fazel et al., 2019) |
|  |  | R | TGTGCGGATGAAGTCAGCTC |  |  |  |
|  | *Sul3* | F | CATTCTAGAAAACAGTCGTAGTTC | 973 | 55 | (Fazel et al., 2019) |
|  |  | R | CATCTGCAGCTAACCTAGGGCTTTGGA |  |  |  |
| Macrolide resistance gene | *mefA* | F | TGTGCATATTTCTATTACG | 1218 | 55 | (Ojo et al., 2004) |
|  |  | R | CCAATTGGCATAGCAAG |  |  |  |
|  | *ereA* | F | GCCGGTGCTCATGAACTTGAG | 419 | 55 | (Tabaran et al., 2017) |
|  |  | R | CGACTCTATTCGATCAGAGGC |  |  |  |
|  | *ereB* | F | GCCTTGAAGCTATGGCTCC | 380 | 55 | (Ojo et al., 2004) |
|  |  | R | GGCCCATTGGTAGGCAAC |  |  |  |
|  | *ermB* | F | GAAAAGGTACTCAACCAAATA | 663 | 55 | (Ojo et al., 2004) |
|  |  | R | AGTAACGGTACTTAAATTGTTTAC |  |  |  |
|  | *mphA* | F | GTGAGGAGGAGCTTCGCGAG | 454 | 55 | (Ojo et al., 2004) |
|  |  | R | TGCCGCAGGACTCGGAGGTC |  |  |  |
|  | *mphB* | F | TTAAACAAGTAATCGAGATAGC | 889 | 55 | (Ojo et al., 2004) |
|  |  | R | CCTTGTACTTCCAATGCTTG |  |  |  |
| Polymyxin resistance gene | *mcr-1* | F | AGTCCGTTTGTTCTTGTGGC | 320 | 55 | (Rebelo et al., 2018) |
|  |  | R | AGATCCTTGGTCTCGGCTTG |  |  |  |
|  | *mcr-2* | F | TATCGCTATGTGCTAAAGCCTG | 816 | 55 | (Jousset et al., 2019) |
|  |  | R | AAAATACTGCGTGGCAGGTAGC |  |  |  |
|  | *mcr-3* | F | AAATAAAAATTGTTCCGCTTATG | 929 | 55 | (Rebelo et al., 2018) |
|  |  | R | AATGGAGATCCCCGTTTTT |  |  |  |
|  | *mcr-4* | F | TCACTTTCATCACTGCGTTG | 1116 | 55 | (Rebelo et al., 2018) |
|  |  | R | TTGGTCCATGACTACCAATG |  |  |  |
|  | *mcr-5* | F | ATGCGGTTGTGCATTTATC | 1644 | 55 | (Rebelo et al., 2018) |
|  |  | R | TCATTGTGGTTGTCCTTTTCTG |  |  |  |
| Phenicol resistance gene | *floR* | F | CACGTTGAGCCTCTATAT | 868 | 55 | (Kuang, 2015) |
|  |  | R | ATGCAGAAGTAGAACGCG |  |  |  |
|  | *cmlA* | F | TGTCATTTACGGCATACTCG | 435 | 55 | (Kuang, 2015) |
|  |  | R | ATCAGGCATCCCATTCCCAT |  |  |  |
|  | *Cat1* | F | AGTTGCTCAATGTACCTATAACC | 547 | 55 | (Jaja et al., 2019) |
|  |  | R | TTGTAATTAAGCATTCTGCC |  |  |  |

^a^ Forward, F; Reverse, R.

^b^ Amplicon length, AL.

^c^ Annealing temperature, AT.

**Table S3** The prevalence of *K. pneumoniae* in different sample types in five farms

| **Type of sample** | **Farm A** | | | **Farm B** | | | **Farm C** | | | **Farm D** | | | **Farm E** | | | **Total** | | |
| --- | --- | --- | --- | --- | --- | --- | --- | --- | --- | --- | --- | --- | --- | --- | --- | --- | --- | --- |
|  | **No. samples** | **No.**  **isolates** | **Proportion (%)** | **No. samples** | **No.**  **isolates** | **Proportion (%)** | **No. samples** | **No.**  **isolates** | **Proportion (%)** | **No. samples** | **No.**  **isolates** | **Proportion (%)** | **No. samples** | **No.**  **isolates** | **Proportion (%)** | **No. samples** | **No.**  **isolates** | **Proportion (%)** |
| Nipple milk of healthy cow | 8 | 1 | 12.50 | 8 | 1 | 12.50 | 31 | 10 | 32.26 | 12 | 7 | 58.33 | 37 | 5 | 13.51 | 96 | 24 | 25.00 |
| Nipple milk of SCM cow | 2 | 0 | 0.00 | 9 | 2 | 22.22 | 19 | 4 | 21.05 | 8 | 5 | 62.50 | 11 | 1 | 9.09 | 49 | 12 | 24.49 |
| Nipple milk of CM cow | 19 | 3 | 15.79 | 24 | 2 | 8.33 | 43 | 20 | 46.51 | 52 | 10 | 19.23 | 0 | 0 | 0.00 | 138 | 35 | 25.36 |
| Tank-milk | 2 | 0 | 0.00 | 1 | 0 | 0.00 | 3 | 0 | 0.00 | 0 | 0 | 0.00 | 1 | 1 | 100.00 | 7 | 1 | 14.29 |
| Nipple swab of healthy cow | 8 | 1 | 12.50 | 19 | 5 | 26.32 | 31 | 13 | 41.94 | 0 | 0 | 0.00 | 37 | 3 | 8.11 | 95 | 22 | 23.16 |
| Nipple swab of SCM cow | 2 | 0 | 0.00 | 7 | 3 | 42.86 | 19 | 10 | 52.63 | 0 | 0 | 0.00 | 11 | 0 | 0.00 | 39 | 13 | 33.33 |
| Nipple swab of CM cow | 18 | 4 | 22.22 | 22 | 6 | 27.27 | 38 | 13 | 34.21 | 0 | 0 | 0.00 | 0 | 0 | 0.00 | 78 | 23 | 29.49 |
| Anal swab of healthy cow | 8 | 0 | 0.00 | 21 | 2 | 9.52 | 31 | 12 | 38.71 | 0 | 0 | 0.00 | 37 | 9 | 24.32 | 97 | 23 | 23.71 |
| Anal swab of SCM cow | 2 | 0 | 0.00 | 1 | 0 | 0.00 | 19 | 5 | 26.32 |  |  |  | 11 | 3 | 27.27 | 33 | 8 | 24.24 |
| Anal swab of CM cow | 18 | 2 | 11.11 | 24 | 6 | 25.00 | 38 | 13 | 34.21 | 0 | 0 | 0.00 | 0 | 0 | 0.00 | 80 | 21 | 26.25 |
| Milk cup swab | 2 | 0 | 0.00 | 5 | 2 | 40.00 | 7 | 1 | 14.29 | 0 | 0 | 0.00 | 5 | 0 | 0.00 | 19 | 3 | 15.79 |
| Feed from the health bar | 2 | 1 | 50.00 | 7 | 1 | 14.29 | 7 | 2 | 28.57 | 0 | 0 | 0.00 | 7 | 7 | 100.00 | 23 | 11 | 47.83 |
| Feed from the CM bar | 2 | 1 | 50.00 | 1 | 0 | 0.00 | 3 | 1 | 33.33 | 0 | 0 | 0.00 | 0 | 0 | 0.00 | 6 | 2 | 33.33 |
| Feed stored in the warehouse |  |  |  | 1 | 1 | 100.00 | 1 | 0 | 0.00 | 0 | 0 | 0.00 | 1 | 0 | 0.00 | 3 | 1 | 33.33 |
| Bedding from the healthy bar | 2 | 1 | 50.00 | 7 | 2 | 28.57 | 7 | 0 | 0.00 | 0 | 0 | 0.00 | 7 | 5 | 71.43 | 23 | 8 | 34.78 |
| Bedding from the CM bar | 2 | 1 | 50.00 | 1 | 0 | 0.00 | 3 | 1 | 33.33 | 0 | 0 | 0.00 | 0 | 0 | 0.00 | 6 | 2 | 33.33 |
| Beeding stored in the warehouse |  |  |  | 1 | 0 | 0.00 | 1 | 0 | 0.00 | 0 | 0 | 0.00 | 1 | 1 | 100.00 | 3 | 1 | 33.33 |
| Faeces from the healthy bar | 2 | 1 | 50.00 | 7 | 2 | 28.57 | 7 | 2 | 28.57 | 0 | 0 | 0.00 | 7 | 6 | 85.71 | 23 | 11 | 47.83 |
| Faeces from the CM bar | 2 | 1 | 50.00 | 1 | 0 | 0.00 | 3 | 0 | 0.00 | 0 | 0 | 0.00 | 0 | 0 | 0.00 | 6 | 1 | 16.67 |
| Air | 2 | 1 | 50.00 | 9 | 2 | 22.22 | 8 | 5 | 62.50 | 0 | 0 | 0.00 | 7 | 1 | 14.29 | 26 | 9 | 34.62 |
| Drinking water | 2 | 0 | 0.00 | 1 | 0 | 0.00 | 8 | 1 | 12.50 | 0 | 0 | 0.00 | 7 | 5 | 71.43 | 18 | 6 | 33.33 |
| Spray water | 2 | 0 | 0.00 | 1 | 0 | 0.00 | 8 | 1 | 12.50 | 0 | 0 | 0.00 | 1 | 1 | 100.00 | 12 | 2 | 16.67 |
| Washing water | 2 | 0 | 0.00 | 1 | 0 | 0.00 | 3 | 0 | 0.00 | 0 | 0 | 0.00 | 1 | 0 | 0.00 | 7 | 0 | 0.00 |
| Total | 109 | 18 | 16.51^a^ | 179 | 37 | 20.67^ab^ | 338 | 114 | 33.73^c^ | 72 | 22 | 30.56^bc^ | 189 | 48 | 25.40^abd^ | 887 | 239 | 26.94 |

Note: The different letter (a/b/c/d) indicated significant difference, and the same letter indicate no significant difference.

**Table S4** MIC distribution of 239 *K. pneumoniae* to 17 antimicrobials

| **Antimicrobials** | **Range of antibiotic concentration (µg/mL）** | **Number of isolates with different MICs** | | | | | | | | | | | | | | **S** | **I** | **R** | **MIC_50_**  **(µg/mL)** | **MIC_90_**  **(µg/mL)** |
| --- | --- | --- | --- | --- | --- | --- | --- | --- | --- | --- | --- | --- | --- | --- | --- | --- | --- | --- | --- | --- |
|  |  | **0.125** | **0.25** | **0.5** | **1** | **2** | **4** | **8** | **16** | **32** | **64** | **128** | **256** | **512** | **1024** | **%** | **%** | **%** |  |  |
| AMP | 0.25~128 |  |  |  | 1 | 1 |  | 2 | 14 | 67 | 83 | 71^a^ |  |  |  | 0 | 0 | 100 | 64 | ＞128 |
| AMC | 0.25/0.12~128/64 |  | 5^b^ | 13 | 112 | 47 | 16 | 21 | 19 | 4 | 1 | 1^a^ |  |  |  | 89.54 | 7.95 | 2.51 | 1/0.5 | 16/8 |
| CEF^c^ | 0.25~128 |  | 15^b^ |  | 5 | 18 | 88 | 57 | 16 | 11 | 4 | 25^a^ |  |  |  | 15.90 | 36.82 | 47.28 | 4 | 128 |
| EFT | 0.25~128 |  | 68^b^ | 101 | 33 | 3 | 3 | 3 | 7 | 5 | 1 | 15^a^ |  |  |  | 85.77 | 1.26 | 12.97 | 0.5 | 16 |
| CRO | 0.25~128 |  | 201^b^ | 1 | 1 | 1 |  | 3 | 6 | 4 | 4 | 18^a^ |  |  |  | 84.94 | 0.42 | 14.64 | ≤0.25 | 32 |
| MEM | 0.25~128 |  | 237^b^ |  |  | 1 |  | 1 |  |  |  |  |  |  |  | 99.16 | 0.42 | 0.42 | ≤0.25 | ≤0.25 |
| STR^d^ | 0.25~128 |  | 28^b^ | 4 | 16 | 77 | 14 | 6 | 9 | 13 | 13 | 59^a^ |  |  |  | 64.44 | 5.44 | 30.13 | 2 | >128 |
| KAN | 0.5~256 |  |  | 30^b^ | 86 | 45 | 29 | 6 | 3 | 4 | 10 | 5 | 21^a^ |  |  | 83.26 | 1.67 | 15.06 | 2 | 128 |
| GEN | 0.25~128 |  | 138^b^ | 24 | 5 | 10 | 6 | 4 | 19 | 24 | 6 | 3^a^ |  |  |  | 76.57 | 1.67 | 21.76 | ≤0.25 | 32 |
| TET | 0.25~128 |  | 2^b^ | 3 | 64 | 77 | 28 | 5 | 3 | 9 | 22 | 26^a^ |  |  |  | 72.80 | 2.09 | 25.10 | 2 | 128 |
| DOX | 0.25~128 |  | 18^b^ | 2 | 20 | 62 | 16 | 72 | 38 | 10 | 1 |  |  |  |  | 49.37 | 30.13 | 20.50 | 8 | 16 |
| FFC | 0.25~128 |  | 6^b^ | 1 | 3 | 83 | 82 | 16 | 6 | 3 | 2 | 38^a^ |  |  |  | 73.22 | 6.69 | 20.50 | 4 | >128 |
| CT | 0.25~128 |  | 112^b^ | 36 | 74 | 16 | 1 |  |  |  |  |  |  |  |  | 99.58 | 0 | 0.42 | 0.5 | 1 |
| RFX^e^ | 0.25~128 |  | 2^b^ |  | 2 | 2 |  | 4 | 28 | 89 | 73 | 39^a^ |  |  |  | - | - | 16.32 | 32 | 128 |
| CIP | 0.25~128 |  | 169^b^ | 41 | 10 | 6 | 5 | 8 | 1 |  |  |  |  |  |  | 70.71 | 17.15 | 12.55 | ≤0.25 | 1 |
| SOX | 2~1024 |  |  |  |  |  |  | 2 | 1 | 1 |  | 6 | 3 | 2 | 224^a^ | 5.44 | 0 | 94.56 | >1024 | >1024 |
| SXT | 0.12/2.4~64/1216 | 121^b^ | 45 | 17 | 7 | 1 | 2 | 1 | 3 |  | 42^a^ |  |  |  |  | 79.92 | 0 | 20.08 | ≤0.12/2.4 | 64/1216 |

Note: AMP=ampicillin; AMC=amoxicillin/clavulanic acid; CEF=cephalothin; EFT=ceftiofur; CRO=ceftriaxone; MEM=meropenem; STR=streptomycin; KAN=kanamycin; GEN=gentamicin; TET=tetracycline; DOX=doxycycline; FFC=florfenicol; CT=colistin; RFX=rifaximin; CIP=ciprofloxacin; SOX=sulfisoxazole; SXT=sulfamethoxazole/trimethoprim.

The left side of the single vertical line (“│”) in the distribution of MICs indicates the sensitivity range.

The right side of the double vertical line (“││”) in the distribution of MICs indicates the resistance range.

a: Including higher than this tested concentration MIC value.

b: Including lower than this tested concentration MIC value.

c: No CLSI interpretative criteria for cephalothin currently available, refer to resistance breakpoint of ceftiofur.

d: No CLSI interpretative criteria for streptomycin currently available, refer to resistance breakpoint of gentamicin.

e: No CLSI interpretative criteria for rifaximin currently available, refer to the establishment of the critical value of wild strains.

S: Sensitive; I: Intermediary; R: Resistance.

**Table S5** The frequency of the phenotypic multidrug-resistance and the antibiotic-resistance genes among the retrieved strains (n = 105)

| **No. of strains** | **%** | **Type of resistance** | **Phenotypic multidrug resistance** | **The antibiotic resistance genes** |
| --- | --- | --- | --- | --- |
| 11 | 10.4 | MDR | 1st and 2nd generation cephalosporins: cephalothin  Aminoglycosides: streptomycin, gentamicin  Sulfonamides: sulfisoxazole | *blaTEM, blaSHV, strA, strB, aacC2, sul1, sul2, qnrB, ereA, mphA* (n=1)  *blaTEM, blaSHV, strA, strB, aadA1, aacC2, sul1, sul2, qnrB, mphA* (n=1)  *blaTEM, blaSHV, strA, strB, aadA1, aacC2, aacA4, sul1, sul2, qnrB, qnrD, mphA* (n=1)  *blaTEM, blaOXA-1, strA, aadA1, aadA2, aacC2, aacA4, sul1, sul2, sul3, aac(6')-Ib-cr, qnrS, tetA, floR, cmlA* (n=1)  *blaTEM, blaSHV, strA, strB, aacC2, sul1, sul2, qnrB, mphA* (n=1)  *blaTEM, blaSHV, strA, strB, aadA1, aacC2, aacA4, sul1, sul2, qnrB, mphA, floR* (n=2)  *blaTEM, strA, strB, aadA1, aacC2, aacA4, sul1, sul2, qnrB, mphA, floR* (n=1)  *blaTEM, blaSHV, strA, strB, aadA1, aacC2, sul1, sul2, qnrB, mphA, floR* (n=1)  *blaTEM, blaSHV, strA, strB, aacC2, sul1, sul2, qnrB, mphA, cmlA* (n=1)  *blaTEM, blaSHV, strA, strB, aacC2, sul1, sul2, aac(6')-Ib-cr, qnrB, mphA* (n=1) |
| 4 | 3.81 | MDR | 1st and 2nd generation cephalosporins: cephalothin  Aminoglycosides: gentamicin  Tetracyclines: tetracycline, doxycycline  Amphenicols: florfenicol  Sulfonamides: sulfisoxazole, sulfamethoxazole/trimethoprim | blaTEM, blaOXA-1, strB, aadA1, aadA2, aacC2, aacA4, sul1, sul2, sul3, aac(6')-Ib-cr, qnrS, tetA, mphA, floR, cmlA (n=1)  blaTEM, blaSHV, strA, aadA1, aadA2, aacC2, sul2, sul3, qnrS, tetA, floR, cmlA (n=1)  blaTEM, strA, strB, aadA1, aadA2, aacC2, sul2, sul3, qnrS, tetA, floR, cmlA (n=1)  blaTEM, aadA1, aadA2, aacC2, sul2, sul3, qnrS, tetA, floR, cmlA (n=1) |
| 4 | 3.81 | MDR | Tetracyclines: tetracycline, doxycycline  Amphenicols: florfenicol  Sulfonamides: sulfisoxazole, sulfamethoxazole/trimethoprim | *blaTEM, strB, aadA1, sul2, aac(6')-Ib-cr, qnrS, tetA, floR* (n=1)  *blaTEM, blaSHV, strA, strB, aadA1, sul1, aac(6')-Ib-cr, qnrB, qnrS, tetA, ermB, floR* (n=1)  *blaTEM, blaSHV, strB, aadA1, aac(6')-Ib-cr, qnrS, tetA, floR* (n=1)  *blaTEM, blaSHV, aac(6')-Ib-cr* (n=1) |
| 3 | 2.86 | MDR | 1st and 2nd generation cephalosporins: cephalothin  3st and 4nd generation cephalosporins: ceftiofur  Sulfonamides: sulfisoxazole | blaTEM, blaSHV, strA, strB, aadA1, aac(6')-Ib-cr (n=1)  blaTEM, aadA1, aac(6')-Ib-cr (n=1)  blaTEM, aadA1, qnrS, ermB (n=1) |
| 3 | 2.86 | MDR | 1st and 2nd generation cephalosporins: cephalothin  Aminoglycosides: streptomycin, gentamicin  Sulfonamides: sulfisoxazole  Rifamycins: rifaximin | blaTEM, blaSHV, strA, strB, aacC2, sul1, sul2, qnrB, mphA (n=2)  blaTEM, blaSHV, strA, strB, aadA1, aacC2, sul1, sul2, qnrB, mphA, floR (n=1) |
| 3 | 2.86 | MDR | Tetracyclines: tetracycline, doxycycline  Amphenicols: florfenicol  Fluoroquinolones: ciprofloxacin  Sulfonamides: sulfisoxazole, sulfamethoxazole/trimethoprim  Rifamycins: rifaximin | blaTEM, blaSHV, strB, aadA1, aacA4, sul1, aac(6')-Ib-cr, qnrB, tetA, mphA, ermB, floR, cmlA (n=1)  blaTEM, blaSHV, strB, aadA1, aac(6')-Ib-cr (n=1)  blaTEM, blaSHV, aacA4, sul1, aac(6')-Ib-cr, tetD, mphA, floR (n=1) |
| 3 | 2.86 | MDR | 1st and 2nd generation cephalosporins: cephalothin  3st and 4nd generation cephalosporins: ceftriaxone  Aminoglycosides: streptomycin, gentamicin, kanamycin  Sulfonamides: sulfisoxazole | blaTEM, blaSHV, strA, strB, aadA1, aacC2, aacA4, sul1, aac(6')-Ib-cr, qnrB, mphA, floR (n=1)  CTX-M-14, blaTEM, strA, strB, aadA1, aacC2, aacA4, aphA1, sul2, aac(6')-Ib-cr, qnrS (n=1)  blaTEM, blaSHV, strB, aadA1, aadA2, aac(6')-Ib-cr, ermB (n=1) |
| 2 | 1.90 | MDR | 1st and 2nd generation cephalosporins: cephalothin  Fluoroquinolones: ciprofloxacin  Sulfonamides: sulfisoxazole | blaTEM, blaSHV, strA, aadA1, aac(6')-Ib-cr (n=1)  blaTEM, strA, aadA1 (n=1) |
| 2 | 1.90 | MDR | 1st and 2nd generation cephalosporins: cephalothin  3st and 4nd generation cephalosporins: ceftriaxone  Aminoglycosides: streptomycin, gentamicin  Sulfonamides: sulfisoxazole | *blaTEM, blaSHV, strA, strB, aacC2, sul1, sul2, qnrB, mphA* (n=2) |
| 2 | 1.90 | MDR | 1st and 2nd generation cephalosporins: cephalothin  Aminoglycosides: gentamicin  Tetracyclines: tetracycline, doxycycline  Amphenicols: florfenicol  Sulfonamides: sulfisoxazole, sulfamethoxazole/trimethoprim  Rifamycins: rifaximin | blaTEM, aadA1, aadA2, aacC2, sul2, sul3, qnrS, tetA, floR, cmlA (n=1)  blaTEM, strA, aadA1, aadA2, aacC2, sul2, sul3, qnrS, tetA, floR, cmlA (n=1) |
| 2 | 1.90 | MDR | 1st and 2nd generation cephalosporins: cephalothin  Aminoglycosides: kanamycin  Tetracyclines: tetracycline, doxycycline  Amphenicols: florfenicol  Fluoroquinolones: ciprofloxacin  Sulfonamides: sulfisoxazole, sulfamethoxazole/trimethoprim  Rifamycins: rifaximin | blaTEM, blaSHV, blaOXA-1, strB, aadA1, aacA4, sul1, aac(6')-Ib-cr, tetA, mphA, ermB, floR (n=1)  blaTEM, strA, aadA1, aacA4, aac(6')-Ib-cr, qnrB, tetA, mphA, ermB, floR (n=1) |
| 2 | 1.90 | MDR | 1st and 2nd generation cephalosporins: cephalothin  3st and 4nd generation cephalosporins: ceftriaxone  Aminoglycosides: streptomycin  Tetracyclines: tetracycline, doxycycline  Sulfonamides: sulfisoxazole | CTX-M-15, blaTEM, blaSHV, strA, strB, sul2, aac(6')-Ib-cr, qnrB, tetA, mphA (n=2) |
| 2 | 1.90 | MDR | 1st and 2nd generation cephalosporins: cephalothin  Aminoglycosides: streptomycin, gentamicin, kanamycin  Tetracyclines: tetracycline, doxycycline  Amphenicols: florfenicol  Fluoroquinolones: ciprofloxacin  Sulfonamides: sulfisoxazole, sulfamethoxazole/trimethoprim  Rifamycins: rifaximin | blaTEM, blaSHV, blaOXA-1, strA, strB, aadA1, aacC2, aacA4, aphA1, sul1, sul2, aac(6')-Ib-cr, qnrB, tetA, mphA, ermB, floR (n=1)  blaTEM, blaSHV, blaOXA-1, strA, strB, aadA1, aacC2, aacA4, aphA1, sul1, sul2, aac(6')-Ib-cr, tetA, mphA, ermB, floR (n=1) |
| 2 | 1.90 | MDR | 1st and 2nd generation cephalosporins: cephalothin  Aminoglycosides: streptomycin, kanamycin  Tetracyclines: tetracycline, doxycycline  Amphenicols: florfenicol  Fluoroquinolones: ciprofloxacin  Sulfonamides: sulfisoxazole, sulfamethoxazole/trimethoprim  Rifamycins: rifaximin | blaTEM, blaSHV, blaOXA-1, strA, strB, aadA1, aacA4, sul1, aac(6')-Ib-cr, qnrB, tetA, mphA, ermB, floR (n=1)  blaTEM, blaSHV, strB, aadA1, aacA4, aphA1, sul1, sul2, aac(6')-Ib-cr, qnrB, tetA, mphA, ermB, floR (n=1) |
| 2 | 1.90 | MDR | 1st and 2nd generation cephalosporins: cephalothin  Aminoglycosides: streptomycin  Sulfonamides: sulfisoxazole | blaTEM, blaSHV, strA, strB, aadA1, aac(6')-Ib-cr, ermB (n=2) |
| 2 | 1.90 | MDR | 1st and 2nd generation cephalosporins: cephalothin  Tetracyclines: tetracycline, doxycycline  Amphenicols: florfenicol  Sulfonamides: sulfisoxazole | blaTEM, blaSHV, strA, strB, aadA1, sul2, tetA, mphA, ermB, floR  blaTEM, strA, strB, aadA1, floR (n=1) |
| 2 | 1.90 | MDR | 1st and 2nd generation cephalosporins: cephalothin  Tetracyclines: tetracycline, doxycycline  Sulfonamides: sulfisoxazole | blaTEM, blaSHV, aadA1 (n=1)  blaTEM, blaSHV, strA, strB, aadA1, aac(6')-Ib-cr, tetA, mphA (n=1) |
| 2 | 1.90 | MDR | Aminoglycosides: kanamycin  Tetracyclines: tetracycline, doxycycline  Amphenicols: florfenicol  Fluoroquinolones: ciprofloxacin  Sulfonamides: sulfisoxazole, sulfamethoxazole/trimethoprim  Rifamycins: rifaximin | blaTEM, blaSHV, blaOXA-1, strB, aacA4, sul1, aac(6')-Ib-cr, qnrB, tetA, ermB, floR (n=1)  blaTEM, blaOXA-1, strB, aadA1, aacA4, sul1, aac(6')-Ib-cr, tetA, mphA, floR (n=1) |
| 2 | 1.90 | MDR | Aminoglycosides: streptomycin, gentamicin  Sulfonamides: sulfisoxazole  Rifamycins: rifaximin | blaTEM, blaSHV, strA, strB, aacC2, sul1, sul2, qnrB, mphA (n=1)  blaTEM, blaSHV, strA, strB, aadA1, aacC2, sul1, sul2, qnrB, mphA, floR (n=1) |
| 2 | 1.90 | MDR | Aminoglycosides: streptomycin  Tetracyclines: tetracycline, doxycycline  Amphenicols: florfenicol  Sulfonamides: sulfisoxazole, sulfamethoxazole/trimethoprim  Rifamycins: rifaximin | blaTEM, blaSHV, strA, strB, aadA1, sul2, aac(6')-Ib-cr, qnrS, tetA, ermB, floR, cmlA (n=1)  blaTEM, strA, strB, aadA1, aac(6')-Ib-cr, tetA, floR, cmlA (n=1) |
| 1 | 0.95 | MDR | 1st and 2nd generation cephalosporins: cephalothin  Fluoroquinolones: ciprofloxacin  Sulfonamides: sulfisoxazole  Rifamycins: rifaximin | blaTEM, blaSHV, aadA1, aacA4, floR (n=1) |
| 1 | 0.95 | MDR | 1st and 2nd generation cephalosporins: cephalothin  Sulfonamides: sulfisoxazole  Rifamycins: rifaximin | blaTEM, blaSHV, aadA1, aacA4 (n=1) |
| 1 | 0.95 | MDR | 1st and 2nd generation cephalosporins: cephalothin  3st and 4nd generation cephalosporins: ceftriaxone  Sulfonamides: sulfisoxazole | blaTEM, blaSHV, strA, strB, aadA1, aacC2, aacA4, sul1, sul2, qnrB, mphA (n=1) |
| 1 | 0.95 | MDR | 1st and 2nd generation cephalosporins: cephalothin  Aminoglycosides: streptomycin, gentamicin  Fluoroquinolones: ciprofloxacin  Sulfonamides: sulfisoxazole | blaTEM, strA, strB, aadA1, aacC2, sul1, sul2, qnrB, mphA, floR (n=1) |
| 1 | 0.95 | MDR | 1st and 2nd generation cephalosporins: cephalothin  Aminoglycosides: streptomycin  Amphenicols: florfenicol  Sulfonamides: sulfisoxazole  Rifamycins: rifaximin | blaTEM, blaSHV, strA, strB, aadA1, aac(6')-Ib-cr, ermB (n=1) |
| 1 | 0.95 | MDR | 1st and 2nd generation cephalosporins: cephalothin  Aminoglycosides: streptomycin, gentamicin, kanamycin  Sulfonamides: sulfisoxazole | blaTEM, strA, strB, aadA2, aacC2, sul2, aac(6')-Ib-cr, mphA, ermB (n=1) |
| 1 | 0.95 | MDR | Aminoglycosides: streptomycin  Tetracyclines: tetracycline, doxycycline  Fluoroquinolones: ciprofloxacin  Sulfonamides: sulfisoxazole, sulfamethoxazole/trimethoprim | blaTEM, blaSHV, strB, aadA1, aac(6')-Ib-cr, mphA (n=1) |
| 1 | 0.95 | MDR | 1st and 2nd generation cephalosporins: cephalothin  β-Lactam-β-lactamase-inhibitor combinations: amoxicillin/clavulanic acid  Sulfonamides: sulfisoxazole | blaTEM, blaSHV, strB, aadA1, mphA, ermB (n=1) |
| 1 | 0.95 | MDR | 1st and 2nd generation cephalosporins: cephalothin  β-Lactam-β-lactamase-inhibitor combinations: amoxicillin/clavulanic acid  Carbapenems: meropenem  Sulfonamides: sulfisoxazole | blaTEM, blaSHV, strB, aadA1, mphA, ermB (n=1) |
| 1 | 0.95 | MDR | 1st and 2nd generation cephalosporins: cephalothin  3st and 4nd generation cephalosporins: ceftriaxone  β-Lactam-β-lactamase-inhibitor combinations: amoxicillin/clavulanic acid  Sulfonamides: sulfisoxazole | blaTEM, strA, strB, aadA2, sul2, aac(6')-Ib-cr, ermB (n=1) |
| 1 | 0.95 | MDR | Aminoglycosides: kanamycin  Tetracyclines: tetracycline  Amphenicols: florfenicol  Fluoroquinolones: ciprofloxacin  Sulfonamides: sulfisoxazole  Rifamycins: rifaximin | blaTEM, blaSHV, strA, strB, aadA1, aac(6')-Ib-cr, mphA (n=1) |
| 1 | 0.95 | MDR | 1st and 2nd generation cephalosporins: cephalothin  3st and 4nd generation cephalosporins: ceftriaxone  Aminoglycosides: kanamycin  Tetracyclines: tetracycline  Sulfonamides: sulfisoxazole | blaTEM, strA, strB, aacC2, aac(6')-Ib-cr, qnrS (n=1) |
| 1 | 0.95 | MDR | 1st and 2nd generation cephalosporins: cephalothin  Tetracyclines: tetracycline, doxycycline  Amphenicols: florfenicol  Fluoroquinolones: ciprofloxacin  Sulfonamides: sulfisoxazole, sulfamethoxazole/trimethoprim  Rifamycins: rifaximin | blaTEM, blaSHV, strA, strB, aadA1, aacA4, sul1, aac(6')-Ib-cr, qnrB, tetA, mphA, floR (n=1) |
| 1 | 0.95 | MDR | 1st and 2nd generation cephalosporins: cephalothin  Tetracyclines: tetracycline, doxycycline  Amphenicols: florfenicol  Sulfonamides: sulfisoxazole, sulfamethoxazole/trimethoprim | blaTEM, blaSHV, strA, strB, aadA1, sul2, aac(6')-Ib-cr, qnrS, tetA, ermB, floR (n=1) |
| 1 | 0.95 | MDR | β-Lactam-β-lactamase-inhibitor combinations: amoxicillin/clavulanic acid  Aminoglycosides: kanamycin  Tetracyclines: tetracycline, doxycycline  Amphenicols: florfenicol  Fluoroquinolones: ciprofloxacin  Sulfonamides: sulfisoxazole, sulfamethoxazole/trimethoprim  Rifamycins: rifaximin | blaTEM, blaSHV, blaOXA-1, strA, strB, aadA1, aacA4, sul1, aac(6')-Ib-cr, qnrB, tetA, mphA, ermB, floR (n=1) |
| 1 | 0.95 | MDR | β-Lactam-β-lactamase-inhibitor combinations: amoxicillin/clavulanic acid  Aminoglycosides: streptomycin, gentamicin, kanamycin  Tetracyclines: tetracycline, doxycycline  Amphenicols: florfenicol  Fluoroquinolones: ciprofloxacin  Sulfonamides: sulfisoxazole, sulfamethoxazole/trimethoprim  Rifamycins: rifaximin | blaTEM, blaSHV, blaOXA-1, strB, aadA1, aacC2, aacA4, aphA1, sul1, sul2, aac(6')-Ib-cr, tetA, mphA, ermB, floR (n=1) |
| 1 | 0.95 | MDR | 1st and 2nd generation cephalosporins: cephalothin  3st and 4nd generation cephalosporins: ceftriaxone  Aminoglycosides: streptomycin, gentamicin, kanamycin  Sulfonamides: sulfisoxazole, sulfamethoxazole/trimethoprim | blaTEM, blaSHV, strB, aadA1, aac(6')-Ib-cr, ermB (n=1) |
| 1 | 0.95 | MDR | Aminoglycosides: streptomycin, kanamycin  Tetracyclines: tetracycline, doxycycline  Amphenicols: florfenicol  Fluoroquinolones: ciprofloxacin  Sulfonamides: sulfisoxazole, sulfamethoxazole/trimethoprim  Rifamycins: rifaximin | blaTEM, blaSHV, blaOXA-1, strA, strB, aadA1, aacA4, sul1, aac(6')-Ib-cr, tetA, mphA, ermB, floR (n=1) |
| 1 | 0.95 | MDR | 1st and 2nd generation cephalosporins: cephalothin  3st and 4nd generation cephalosporins: ceftriaxone  Aminoglycosides: streptomycin, kanamycin  Amphenicols: florfenicol  Sulfonamides: sulfisoxazole | blaTEM, blaSHV, blaOXA-1, strB, aacA4, sul1, aac(6')-Ib-cr (n=1) |
| 1 | 0.95 | MDR | Tetracyclines: tetracycline  Amphenicols: florfenicol  Sulfonamides: sulfisoxazole | blaTEM, blaSHV, strB, aac(6')-Ib-cr, tetA, ermB (n=1) |
| 1 | 0.95 | MDR | 1st and 2nd generation cephalosporins: cephalothin  3st and 4nd generation cephalosporins: ceftriaxone  Aminoglycosides: gentamicin, kanamycin  Tetracyclines: tetracycline, doxycycline  Fluoroquinolones: ciprofloxacin  Sulfonamides: sulfisoxazole, sulfamethoxazole/trimethoprim | CTX-M-15, blaTEM, blaSHV, blaOXA-1, strA, strB, aacC2, aacA4, sul2, aac(6')-Ib-cr, tetA (n=1) |
| 1 | 0.95 | MDR | 1st and 2nd generation cephalosporins: cephalothin  3st and 4nd generation cephalosporins: ceftriaxone  Aminoglycosides: streptomycin, gentamicin, kanamycin  Sulfonamides: sulfisoxazole  Rifamycins: rifaximin | CTX-M-14, blaTEM, blaSHV, strB, aadA1, aacC2, aphA1, sul2, aac(6')-Ib-cr, qnrS (n=1) |
| 1 | 0.95 | MDR | 1st and 2nd generation cephalosporins: cephalothin  Aminoglycosides: kanamycin  Tetracyclines: tetracycline, doxycycline  Fluoroquinolones: ciprofloxacin  Sulfonamides: sulfisoxazole, sulfamethoxazole/trimethoprim  Rifamycins: rifaximin | blaTEM, blaSHV, blaOXA-1, aacA4, sul1, aac(6')-Ib-cr, tetA, mphA, ermB, floR (n=1) |
| 1 | 0.95 | MDR | 1st and 2nd generation cephalosporins: cephalothin  Aminoglycosides: streptomycin  Tetracyclines: tetracycline, doxycycline  Amphenicols: florfenicol  Fluoroquinolones: ciprofloxacin  Sulfonamides: sulfisoxazole, sulfamethoxazole/trimethoprim | blaTEM, blaSHV, strB, aadA1, aadA2, sul1, sul2, aac(6')-Ib-cr, qnrS, tetA, mphA, floR (n=1) |
|  |  |  | Aminoglycosides: streptomycin  Tetracyclines: tetracycline, doxycycline  Amphenicols: florfenicol  Fluoroquinolones: ciprofloxacin  Sulfonamides: sulfisoxazole, sulfamethoxazole/trimethoprim  Rifamycins: rifaximin | blaTEM, blaSHV, strA, strB, aadA1, qnrS, tetA, floR, cmlA (n=1) |
|  |  |  | 1st and 2nd generation cephalosporins: cephalothin  3st and 4nd generation cephalosporins: ceftiofur, ceftriaxone  Aminoglycosides: streptomycin, kanamycin  Tetracyclines: tetracycline, doxycycline  Sulfonamides: sulfisoxazole, sulfamethoxazole/trimethoprim | CTX-M-15, blaTEM, strA, strB, aadA1, aacA4, aphA1, aac(6')-Ib-cr (n=1) |
|  |  |  | 1st and 2nd generation cephalosporins: cephalothin  3st and 4nd generation cephalosporins: ceftiofur, ceftriaxone  Aminoglycosides: streptomycin, kanamycin  Tetracyclines: tetracycline, doxycycline  Amphenicols: florfenicol  Sulfonamides: sulfisoxazole | strA, aadA1, aac(6')-Ib-cr (n=1) |
|  |  |  | 1st and 2nd generation cephalosporins: cephalothin  3st and 4nd generation cephalosporins: ceftiofur, ceftriaxone  β-Lactam-β-lactamase-inhibitor combinations: amoxicillin/clavulanic acid  Aminoglycosides: streptomycin, kanamycin  Tetracyclines: tetracycline, doxycycline  Amphenicols: florfenicol  Sulfonamides: sulfisoxazole | blaTEM, strA, strB, aadA1, aphA1, sul2, tetA, mphA, floR (n=1) |
|  |  |  | 1st and 2nd generation cephalosporins: cephalothin  3st and 4nd generation cephalosporins: ceftiofur  Fluoroquinolones: ciprofloxacin  Sulfonamides: sulfisoxazole, sulfamethoxazole/trimethoprim | blaTEM, aadA1 (n=1) |
|  |  |  | 1st and 2nd generation cephalosporins: cephalothin  3st and 4nd generation cephalosporins: ceftiofur, ceftriaxone  Aminoglycosides: streptomycin, kanamycin  Tetracyclines: tetracycline, doxycycline  Sulfonamides: sulfisoxazole | CTX-M-15, blaTEM, strA, strB, aadA1, aacA4, aphA1, qnrS, tetA (n=1) |
|  |  |  | 1st and 2nd generation cephalosporins: cephalothin  3st and 4nd generation cephalosporins: ceftiofur, ceftriaxone  Aminoglycosides: streptomycin, kanamycin  Fluoroquinolones: ciprofloxacin  Sulfonamides: sulfisoxazole | CTX-M-15, blaTEM, blaOXA-1, strA, strB, aadA1, aacA4, aphA1, sul2, aac(6')-Ib-cr, qnrS (n=1) |
|  |  |  | 1st and 2nd generation cephalosporins: cephalothin  3st and 4nd generation cephalosporins: ceftiofur, ceftriaxone  Aminoglycosides: streptomycin, kanamycin, gentamicin  Amphenicols: florfenicol  Sulfonamides: sulfisoxazole | blaTEM, strA, strB, aadA1, aphA1, qnrS, floR (n=1) |
|  |  |  | 1st and 2nd generation cephalosporins: cephalothin  3st and 4nd generation cephalosporins: ceftiofur, ceftriaxone  Aminoglycosides: streptomycin, kanamycin, gentamicin  Tetracyclines: tetracycline  Amphenicols: florfenicol  Fluoroquinolones: ciprofloxacin  Sulfonamides: sulfisoxazole | blaTEM, strA, strB, aadA1, aacA4, aphA1, floR (n=1) |
|  |  |  | 1st and 2nd generation cephalosporins: cephalothin  3st and 4nd generation cephalosporins: ceftiofur, ceftriaxone  Aminoglycosides: streptomycin, kanamycin, gentamicin  Tetracyclines: tetracycline, doxycycline  Amphenicols: florfenicol  Sulfonamides: sulfisoxazole | CTX-M-15,blaTEM,blaSHV,strA,strB,aadA1,aacC2,aphA1,sul2,tetA (n=1) |
|  |  |  | 1st and 2nd generation cephalosporins: cephalothin  3st and 4nd generation cephalosporins: ceftiofur, ceftriaxone  Aminoglycosides: streptomycin, kanamycin  Sulfonamides: sulfisoxazole | blaTEM, strA, strB, aadA1, aacC2, aphA1, qnrS, ermB (n=1) |
|  |  |  | 1st and 2nd generation cephalosporins: cephalothin  3st and 4nd generation cephalosporins: ceftiofur, ceftriaxone  Aminoglycosides: streptomycin, kanamycin, gentamicin  Tetracyclines: tetracycline, doxycycline  Sulfonamides: sulfisoxazole | CTX-M-15, blaTEM, strA, strB, aadA1, aphA1, ermB (n=1) |
|  |  |  | 1st and 2nd generation cephalosporins: cephalothin  3st and 4nd generation cephalosporins: ceftiofur, ceftriaxone  Aminoglycosides: streptomycin, gentamicin  Sulfonamides: sulfisoxazole | CTX-M-15, blaTEM, strA, strB, aadA1, aacC2, sul2, qnrB, ermB (n=1) |
|  |  |  | 1st and 2nd generation cephalosporins: cephalothin  Aminoglycosides: streptomycin  Tetracyclines: tetracycline, doxycycline  Sulfonamides: sulfisoxazole  Rifamycins: rifaximin | blaTEM, strA, strB, aadA1, tetB, ermB (n=1) |
|  |  |  | 1st and 2nd generation cephalosporins: cephalothin  3st and 4nd generation cephalosporins: ceftiofur  Fluoroquinolones: ciprofloxacin  Sulfonamides: sulfisoxazole | blaTEM, strA, aadA1, ermB (n=1) |
|  |  |  | 1st and 2nd generation cephalosporins: cephalothin  3st and 4nd generation cephalosporins: ceftiofur, ceftriaxone  Tetracyclines: tetracycline  Sulfonamides: sulfisoxazole | blaTEM, strA, aadA1, aac(6')-Ib-cr (n=1) |
|  |  |  | 1st and 2nd generation cephalosporins: cephalothin  Aminoglycosides: streptomycin  Tetracyclines: tetracycline, doxycycline  Sulfonamides: sulfisoxazole, sulfamethoxazole/trimethoprim | blaTEM, strA, strB, aadA1, sul1, aac(6')-Ib-cr, tetA, ermB (n=1) |
|  |  |  | 1st and 2nd generation cephalosporins: cephalothin  Tetracyclines: tetracycline  Sulfonamides: sulfisoxazole | blaTEM, strA, aadA1 (n=1) |
|  |  |  | 1st and 2nd generation cephalosporins: cephalothin  Tetracyclines: tetracycline  Amphenicols: florfenicol  Sulfonamides: sulfisoxazole, sulfamethoxazole/trimethoprim  Rifamycins: rifaximin | strA, aadA1, aac(6')-Ib-cr, ermB (n=1) |
|  |  |  | Aminoglycosides: kanamycin  Tetracyclines: tetracycline, doxycycline  Sulfonamides: sulfisoxazole, sulfamethoxazole/trimethoprim | strA, aadA1, aadA2, aphA1, aac(6')-Ib-cr, tetA, mphA, ermB (n=1) |
|  |  |  | 1st and 2nd generation cephalosporins: cephalothin  3st and 4nd generation cephalosporins: ceftiofur, ceftriaxone  Sulfonamides: sulfisoxazole | strA, aadA1, aac(6')-Ib-cr, ermB (n=1) |
|  |  |  | Aminoglycosides: kanamycin  Tetracyclines: tetracycline  Amphenicols: florfenicol  Sulfonamides: sulfisoxazole, sulfamethoxazole/trimethoprim  Rifamycins: rifaximin | strA, aadA1, sul1, aac(6')-Ib-cr, ermB (n=1) |
|  |  |  | 1st and 2nd generation cephalosporins: cephalothin  3st and 4nd generation cephalosporins: ceftriaxone  Tetracyclines: tetracycline  Fluoroquinolones: ciprofloxacin  Sulfonamides: sulfisoxazole, sulfamethoxazole/trimethoprim | *strA, aac(6')-Ib-cr, ermB* (n=1) |

Note: MDR: non-susceptible to ≥ one agent in ≥ three antimicrobial classes.

**Table S6** Silent mutations of *gyrA*, *gyrB*, *parC*, *parE* in 30 ciprofloxacin-resistant, 3 ciprofloxacin-intermediate and 7 ciprofloxacin-sensitive *K. pneumoniae*

| **Isolate** | **MIC (µg/mL)** | **DNA gyrase and topoisomerase IV silent mutations ^a^** | | | |
| --- | --- | --- | --- | --- | --- |
|  | **ciprofloxacin** | ***gyrA*** | ***gyrB*** | ***parC*** | ***parE*** |
| K-JX19h21 | 1 | Pro184(CCA→CCG) | Glu351(GAA→GAG); Arg393(CGC→CGT) | – | Val401(CTG→CTA); Leu416(CTT→CTC); Ser451(TCC→TCT); Asp473(GAC→GAT); Ser493(TCC→TCT) |
| K-MJD19c49 | 1 | Pro184(CCA→CCG) | Glu351(GAA→GAG); Ala388(GCC→GCT); Arg393(CGC→CGT); Ala468(GCG→GCC) | – | Ala382(GCG→GCC); Lys400(AAG→AAA); Ser493(TCC→TCT) |
| K-BYS20c107 | 1 | Pro184(CCA→CCG) | Glu351(GAA→GAG); Arg393(CGC→CGT) | – | Val401(CTG→CTA); Leu416(CTT→CTC); Ser451(TCC→TCT); Asp473(GAC→GAT); Ser493(TCC→TCT) |
| K-BYS20b111 | 4 | Pro184(CCA→CCG) | Glu351(GAA→GAG); Asn364(AAC→AAT); Ala388(GCC→GCT); Arg393(CGC→CGT); Ala432(GCT→GCG); Ala468(GCG→GCC) | Leu99(CTG→CTC) | Ala382(GCG→GCC); Leu416(CTT→CTC); Ser451(TCC→TCT); Asp473(GAC→GAT); Cys486(TGT→TGC); Ser493(TCC→TCT) |
| K-MJD20e130 | 8 | Pro184(CCA→CCG) | Glu351(GAA→GAG); Asn364(AAC→AAT); Ala388(GCC→GCT); Arg393(CGC→CGT); Ala432(GCT→GCG); Ala468(GCG→GCC) | Leu99(CTG→CTC) | Ala382(GCG→GCC); Leu416(CTT→CTC); Ser451(TCC→TCT); Asp473(GAC→GAT); Cys486(TGT→TGC); Ser493(TCC→TCT) |
| K-MJD20e133 | 4 | Pro184(CCA→CCG) | Glu351(GAA→GAG); Ala388(GCC→GCT); Arg393(CGC→CGT); Ala442(GCG→GCT); Val453(GTT→GTC) | Leu161(CTG→TTG) | Glu418(GAA→GAG); Ser451(TCC→TCT); Asp473(GAC→GAT) |
| K-MJD20e135 | 1 | Pro184(CCA→CCG) | Glu351(GAA→GAG); Arg393(CGC→CGT) | – | Val401(CTG→CTA); Leu416(CTT→CTC); Ser451(TCC→TCT); Asp473(GAC→GAT); Ser493(TCC→TCT) |
| K-MJD20e137 | 8 | Pro184(CCA→CCG) | Glu351(GAA→GAG); Ala388(GCC→GCT); Arg393(CGC→CGT); Ala432(GCT→GCG); Ala468(GCG→GCC) | Leu99(CTG→CTC) | Ala382(GCG→GCC); Leu416(CTT→CTC); Ser451(TCC→TCT); Asp473(GAC→GAT); Cys486(TGT→TGC); Ser493(TCC→TCT) |
| K-MJD20e141 | 8 | Pro184(CCA→CCG) | Glu351(GAA→GAG); Ala388(GCC→GCT); Arg393(CGC→CGT); Ala432(GCT→GCG); Ala468(GCG→GCC) | Leu99(CTG→CTC) | Ala382(GCG→GCC); Leu416(CTT→CTC); Ser451(TCC→TCT); Asp473(GAC→GAT); Cys486(TGT→TGC); Ser493(TCC→TCT) |
| K-MJD20e144 | 4 | Pro184(CCA→CCG) | Glu351(GAA→GAG); Ala388(GCC→GCT); Arg393(CGC→CGT); Ala432(GCT→GCG); Ala468(GCG→GCC) | Leu99(CTG→CTC) | Ala382(GCG→GCC); Leu416(CTT→CTC); Ser451(TCC→TCT); Asp473(GAC→GAT); Cys486(TGT→TGC); Ser493(TCC→TCT) |
| K-MJD20e148 | 4 | Pro184(CCA→CCG) | Glu351(GAA→GAG); Ala388(GCC→GCT); Arg393(CGC→CGT); Ala432(GCT→GCG); Ala468(GCG→GCC) | Leu161(CTG→TTG) | Glu418(GAA→GAG); Ser451(TCC→TCT); Asp473(GAC→GAT); Ser493(TCC→TCT) |
| K-MJD20e149 | 16 | Pro184(CCA→CCG) | Glu351(GAA→GAG); Ala388(GCC→GCT); Arg393(CGC→CGT); Ala432(GCT→GCG); Ala468(GCG→GCC) | Leu99(CTG→CTC) | Ala382(GCG→GCC); Leu416(CTT→CTC); Ser451(TCC→TCT); Asp473(GAC→GAT); Cys486(TGT→TGC); Ser493(TCC→TCT) |
| K-MJD20e152 | 8 | Pro184(CCA→CCG) | Glu351(GAA→GAG); Ala388(GCC→GCT); Arg393(CGC→CGT); Ala432(GCT→GCG); Ala468(GCG→GCC) | Leu99(CTG→CTC) | Ala382(GCG→GCC); Leu416(CTT→CTC); Ser451(TCC→TCT); Asp473(GAC→GAT); Cys486(TGT→TGC); Ser493(TCC→TCT) |
| K-MJD20c157 | 8 | Pro184(CCA→CCG) | Glu351(GAA→GAG); Ala388(GCC→GCT); Arg393(CGC→CGT); Ala432(GCT→GCG); Ala468(GCG→GCC) | Leu99(CTG→CTC) | Ala382(GCG→GCC); Leu416(CTT→CTC); Ser451(TCC→TCT); Asp473(GAC→GAT); Cys486(TGT→TGC); Ser493(TCC→TCT) |
| K-MJD20c161 | 8 | Pro184(CCA→CCG) | Glu351(GAA→GAG); Ala388(GCC→GCT); Arg393(CGC→CGT); Ala432(GCT→GCG); Ala468(GCG→GCC) | Leu99(CTG→CTC) | Ala382(GCG→GCC); Leu416(CTT→CTC); Ser451(TCC→TCT); Asp473(GAC→GAT); Cys486(TGT→TGC); Ser493(TCC→TCT) |
| K-MJD20c164 | 2 | Pro184(CCA→CCG) | Glu351(GAA→GAG); Ala388(GCC→GCT); Arg393(CGC→CGT) | – | Ala382(GCG→GCC); Leu416(CTT→CTC); Ser451(TCC→TCT); Asp453(GAT→GAC); Val455(GTG→GTT); Asp473(GAC→GAT) |
| K-MJD20c167 | 2 | Pro184(CCA→CCG) | Glu351(GAA→GAG); Ala388(GCC→GCT); Arg393(CGC→CGT) | – | Ser451(TCC→TCT); Asp473(GAC→GAT); Ser493(TCC→TCT) |
| K-MJD20c174 | 8 | Pro184(CCA→CCG) | Glu351(GAA→GAG); Ala388(GCC→GCT); Arg393(CGC→CGT); Ala432(GCT→GCG); Ala468(GCG→GCC); Leu403(CTG→CTA) | Leu99(CTG→CTC) | Ala382(GCG→GCC); Leu416(CTT→CTC); Ser451(TCC→TCT); Asp473(GAC→GAT); Cys486(TGT→TGC); Ser493(TCC→TCT) |
| K-MJD20c178 | 1 | Pro184(CCA→CCG) | Glu351(GAA→GAG); Asn364(AAC→AAT); Val371(GTG→GTA); Ala388(GCC→GCT); Arg393(CGC→CGT) | – | Glu418(GAA→GAG); Ser493(TCC→TCT) |
| K-MJD20c180 | 1 | – | Glu351(GAA→GAG); Asn364(AAC→AAT); Val371(GTG→GTA); Ala388(GCC→GCT); Arg393(CGC→CGT) | Leu161(CTG→TTG); Ile172(ATC→ATA) | Ala382(GCG→GCC); Leu416(CTT→CTC); Ser451(TCC→TCT); Ser493(TCC→TCT) |
| K-MJD20b183 | 4 | Pro184(CCA→CCG) | Glu351(GAA→GAG); Ala388(GCC→GCT); Arg393(CGC→CGT); Ala432(GCT→GCG); Ala468(GCG→GCC) | Leu99(CTG→CTC) | Ser451(TCC→TCT); Asp473(GAC→GAT); Cys486(TGT→TGC); Ser493(TCC→TCT) |
| K-MJD20b194 | 2 | Pro184(CCA→CCG) | Glu351(GAA→GAG); Arg393(CGC→CGT); Leu444(CTG→CTA) | Ile26(ATC→ATT) | Ser451(TCC→TCT); Asp473(GAC→GAT) |
| K-MJD20b197 | 1 | Pro184(CCA→CCG) | Ser347(TCG→TCT); Glu351(GAA→GAG); Ala388(GCC→GCT); Arg393(CGC→CGT) | – | Ser421(TCG→TCT); Ser451(TCC→TCT); Asp473(GAC→GAT); Ser493(TCC→TCT) |
| K-DXH20e222 | 1 | Pro184(CCA→CCG) | Glu351(GAA→GAG); Ala388(GCC→GCT); Arg393(CGC→CGT); Leu444(CTG→CTA) | Ser57(AGC→AGT) | Ala382(GCG→GCC); Leu416(CTT→CTC); Ser451(TCC→TCT); Asp473(GAC→GAT); Ser493(TCC→TCT) |
| K-DXH20e226 | 2 | Pro184(CCA→CCG) | Glu351(GAA→GAG); Asn364(AAC→AAT); Ala388(GCC→GCT); Arg393(CGC→CGT) | – | Ser451(TCC→TCT); Asp473(GAC→GAT) |
| K-DXH20e228 | 1 | Pro184(CCA→CCG) | Glu351(GAA→GAG); Asn364(AAC→AAT); Ala388(GCC→GCT); Arg393(CGC→CGT) | – | Ala382(GCG→GCC); Lys400(AAG→AAA); Ser451(TCC→TCT); Asp473(GAC→GAT) |
| K-QNE20e242 | 2 | Pro184(CCA→CCG) | Ser347(TCG→TCT); Glu351(GAA→GAG); Ala388(GCC→GCT); Arg393(CGC→CGT) | Leu161(CTG→TTG) | Ala382(GCG→GCC); Ser421(TCG→TCT); Ser493(TCC→TCT) |
| K-QNE20e246 | 8 | Pro184(CCA→CCG) | Glu351(GAA→GAG); Ala388(GCC→GCT); Arg393(CGC→CGT); Ala432(GCT→GCG); Ala468(GCG→GCC) | Leu99(CTG→CTC) | Ala382(GCG→GCC); Leu416(CTT→CTC); Ser451(TCC→TCT); Asp473(GAC→GAT); Cys486(TGT→TGC); Ser493(TCC→TCT) |
| K-QNE20g273 | 2 | Pro184(CCA→CCG) | Glu351(GAA→GAG); Arg393(CGC→CGT) | Leu161(CTG→TTG) | Ala376(GCC→GCT); Ala382(GCG→GCC); Leu416(CTT→CTC); Ser451(TCC→TCT); Asp473(GAC→GAT); Ser493(TCC→TCT) |
| K-QNE20WY293 | 1 | Val90(GTG→GTT); Phe109(TTT→TTC);Ala179(GCC→GCG);Asn181(AAC→AAT);Pro184(CCA→CCG); Ile191(ATT→ATC) | Glu351(GAA→GAG); Ala388(GCC→GCT); Arg393(CGC→CGT) | Leu99(CTG→CTC) | Ala372(GCC→GCT); Arg378(CGC→CGT); Ala382(GCG→GCC); Lys384(AAA→AAG); Arg388(CGC→CGT); Ala396(GCG→GCA); Leu397(CTG→TTG); Ala402(GCG→GCA); Cys404(TGT→TGC); Asp408(GAT→GAC); Arg411(CGT→CGC); Leu416(CTT→CTC); Asp420(GAT→GAC); Gly424(GGC→GGT); Pro439(CCG→CCA); Lys441(AAG→AAA); Leu445(CTT→CTG); Ser451(TCC→TCG); Asp473(GAC→GAT); Gly483(GGT→GGC); Ala491(GCG→GCC); Asp492(GAC→GAT); Ser493(TCC→TCT) |
| K-JX19c10 | 0.5 | Pro184(CCA→CCG) | Glu351(GAA→GAG); Asn364(AAC→AAT); Ala388(GCC→GCT); Arg393(CGC→CGT) | Thr178(ACC→ACA) | Ala376(GCC→GCT); Ala382(GCG→GCC); Leu416(CTT→CTC); Ser451(TCC→TCT); Asp473(GAC→GAT) |
| K-MJD19e31 | 0.5 | Val90(GTG→GTT);  Phe109(TTT→TTC);Ala179(GCC→GCG);Asn181(AAC→AAT);Pro184(CCA→CCG);  Ile191(ATT→ATC) | Ser343(TCC→TCT);  Lys346(AAA→AAG);  Ser347(TCG→TCT);  Val349(GTG→GTA);  Glu350(GAG→GAA);  Glu351(GAA→GAG);  Ala368(GCG→GCC);  Glu390(GAG→GAA);  Thr392(ACT→ACC);  Glu412(GAG→GAA);  Ser418(TCC→TAT);  Leu422(CTG→CTC);  Arg438(CGC→CGT);  Leu451(CTT→CTC);  Ala473(GCG→GCC);  Leu474(CTG→CTT) | Tyr47(TAT→TAC); Arg96(CGC→CGT); Tyr97(TAT→TAC);  Val100(GTG→GTA); Asp101(GAT→GAC);  Gly102(GGT→GGG);  Gly107(GGG→GGT);  Pro112(CCC→CCG); Ser114(TCT→TCC);  Glu122(GAA→GAG); Ala129(GCC→GCT); Glu130(GAG→GAA); Glu135(GAG→GAA); Gly137(GGG→GGA); Gly139(GGG→GGA); Val144(GTG→GTA); Gly149(GGC→GGG);Pro158(CCA→CCT); Leu161(CTG→CTA);  Leu166(CTG→CTT);  Asn167(AAC→AAT);  Val174(GTA→GTG);  Gly175(GGC→GGT);  Ala177(GCG→GCC);  Thr178(ACC→ACT) | Ala372(GCC→GCT);  Arg378(CGC→CGT);  Ala382(GCG→GCC);  Lys384(AAA→AAG);  Arg388(CGC→CGT);  Ala396(GCG→GCA);  Leu397(CTG→TTG);  Ala402(GCG→GCA);  Cys404(TGT→TGC);  Asp408(GAT→GAC);  Arg411(CGT→CGC);  Leu416(CTT→CTC);  Asp420(GAT→GAC);  Gly424(GGC→GGT);  Lys441(AAG→AAA);  Leu445(CTT→CTG);  Ser451(TCC→TCG);  Asp473(GAC→GAT);  Gly483(GGT→GGC);  Ala491(GCG→GCC);  Asp492(GAC→GAT) |
| K-MJD19e33 | 0.5 | Pro184(CCA→CCG) | Glu351(GAA→GAG);  Asn364(AAC→AAT);  Ala388(GCC→GCT);  Arg393(CGC→CGT) | Thr178(ACC→ACA) | Ala376(GCC→GCT);  Ala382(GCG→GCC);  Leu416(CTT→CTC);  Ser451(TCC→TCT);  Asp473(GAC→GAT) |
| K-JX19e02 | ≤0.25 | Pro184(CCA→CCG) | Ala388(GCC→GCT);  Arg393(CGC→CGT);  Ala468(GCG→GCC) | – | Ala382(GCG→GCC);  Lys400(AAG→AAA) |
| K-JX19b13 | ≤0.25 | Pro184(CCA→CCG) | Ala388(GCC→GCT);  Arg393(CGC→CGT);  Ala468(GCG→GCC) | – | Ala382(GCG→GCC);  Lys400(AAG→AAA);  Ser493(TCC→TCT) |
| K-MJD19e34 | ≤0.25 | Pro184(CCA→CCG) | Ala388(GCC→GCT);  Arg393(CGC→CGT);  Ala468(GCG→GCC) | – | Ala382(GCG→GCC);  Lys400(AAG→AAA);  Ser493(TCC→TCT) |
| K-MJD19b50 | ≤0.25 | Pro164(CCT→CCC);  Pro184(CCA→CCG) | Glu351(GAA→GAG) | – | Val401(CTG→CTA);  Leu416(CTT→CTC);  Gly424(GGC→GGT);  Ser451(TCC→TCT);  Asp473(GAC→GAT);  Ser493(TCC→TCT) |
| K-MJD19b56 | ≤0.25 | Pro184(CCA→CCG) | Ala388(GCC→GCT);  Arg393(CGC→CGT);  Ala468(GCG→GCC) | – | Ala382(GCG→GCC);  Lys400(AAG→AAA);  Ser493(TCC→TCT) |
| K-BYS20c105 | ≤0.25 | Pro184(CCA→CCG) | Glu351(GAA→GAG);  Ala388(GCC→GCT);  Arg393(CGC→CGT) | – | – |
| K-DXH20e214 | ≤0.25 | Pro184(CCA→CCG) | Glu351(GAA→GAG);  Arg393(CGC→CGT) | – | Glu418(GAA→GAG);  Ser451(TCC→TCT);  Asp473(GAC→GAT) |

^a^ – Indicates isolates with no mutations.

**REFERENCE**

Almaghrabi, R., Clancy, C. J., Doi, Y., Hao, B., Chen, L., Shields, R. K., et al. (2014). Carbapenem-resistant *Klebsiella pneumoniae* strains exhibit diversity in aminoglycoside-modifying enzymes, which exert differing effects on plazomicin and other agents. *Antimicrob Agents Chemother*. 58 (8): 4443–4451. doi: 10.1128/AAC.00099-14.

Al-Marzooq, F., Ngeow, Y. F., and Tay, S. T. (2015). Emergence of *Klebsiella pneumoniae* producing dual carbapenemases (*NDM-1* and *OXA-232*) and 16S rRNA methylase (*armA*) isolated from a Malaysian patient returning from India. *Int J Antimicrob Agents.* 45, 445–446. doi:10.1016/j.ijantimicag.2014.12.013.

Andrade, L. N., Novais, Â., Stegani, L. M. M., Ferreira, J. C., Rodrigues, C., Darini, A. L. C., et al. (2018). Virulence genes, capsular and plasmid types of multidrug-resistant *CTX-M*(-2, -8, -15) and *KPC*-2-producing *Klebsiella pneumoniae* isolates from four major hospitals in Brazil. *Diagn Microbiol Infect Dis.* 91, 164–168. doi:10.1016/j.diagmicrobio.2018.01.007.

Candan, E. D., and Aksöz, N. (2015). *Klebsiella pneumoniae*: Characteristics of carbapenem resistance and virulence factors. *Acta Biochim Pol.* 62, 867–874. doi:10.18388/abp.2015_1148.

Chuong, L. Van, Prachayasittikul, V., Isarankura Na Ayudhya, C., and Lawung, R. (2018). Multiplex PCR scheme for variant plasmid mediated class C β-lactamase typing. *J Clin Lab Anal.* 32, e22298. doi:10.1002/jcla.22298.

Derakhshan, S., Peerayeh, S. N., and Bakhshi, B. (2016). Association between presence of virulence genes and antibiotic resistance in clinical *Klebsiella pneumoniae* isolates.  *Lab Med.* 47, 306–311. doi:10.1093/labmed/lmw030.

Fadare, F. T., Adefisoye, M. A., and Okoh, A. I. (2020). Occurrence, identification, and antibiogram signatures of selected Enterobacteriaceae from Tsomo and Tyhume rivers in the Eastern Cape Province, Republic of South Africa. *PLoS One.* 15, 1–27. doi:10.1371/journal.pone.0238084.

Fazel, F., Jamshidi, A., and Khoramian, B. (2019). Phenotypic and genotypic study on antimicrobial resistance patterns of *E. coli* isolates from bovine mastitis. *Microb Pathog.* 132, 355–361. doi:10.1016/j.micpath.2019.05.018.

Gharout-Sait, A., Alsharapy, S. A., Brasme, L., Touati, A., Kermas, R., Bakour, S., et al. (2014). Enterobacteriaceae isolates carrying the New Delhi metallo-β-lactamase gene in Yemen. *J Med Microbiol.* 63, 1316–1323. doi:10.1099/jmm.0.073767-0.

Granier, S. A., Hidalgo, L., Millan, A. S., Escudero, J. A., Gutierrez, B., Brisabois, A., et al. (2011). *ArmA* methyltransferase in a monophasic *Salmonella enterica* isolate from food. *Antimicrob Agents Chemother.* 55, 5262–5266. doi:10.1128/AAC.00308-11.

Harada, S., and Doia, Y. (2018). Hypervirulent *Klebsiella pneumoniae*: A call for consensus definition and international collaboration. *J Clin Microbiol.* 56, e00959-18. doi:10.1128/JCM.00959-18.

He, J. (2012). Study on serotypes and distribution characteristic of virulence genes of *Klebsiella Pneumoniae*. *ChongQing Medical University*.

Huang, S., Dai, W., Sun, S., Zhang, X., and Zhang, L. (2012). Prevalence of Plasmid-Mediated Quinolone Resistance and Aminoglycoside Resistance Determinants among Carbapeneme Non-Susceptible Enterobacter cloacae. *PLoS One.* 7, e47636. doi:10.1371/journal.pone.0047636.

Jaja, I. F., Bhembe, N. L., Green, E., Oguttu, J., and Muchenje, V. (2019). Molecular characterisation of antibiotic-resistant *Salmonella enterica* isolates recovered from meat in South Africa. *Acta Trop.*  190, 129–136. doi:10.1016/j.actatropica.2018.11.003.

Jousset, A. B., Bernabeu, S., Bonnin, R. A., Creton, E., Cotellon, G., Sauvadet, A., et al. (2019). Development and validation of a multiplex polymerase chain reaction assay for detection of the five families of plasmid-encoded colistin resistance. *Int J Antimicrob Agents.* 53, 302–309. doi:10.1016/j.ijantimicag.2018.10.022.

Kuang, X. (2015). Prevalence and molecular characteristic of resistance and virulence of three different livestock pathogen bacteria in central China. *HuaZhong Agricultural University*.

Lan, Y., Zhou, M., Jian, Z., Yan, Q., Wang, S., and Liu, W. (2019). Prevalence of pks gene cluster and characteristics of *Klebsiella pneumoniae*-induced bloodstream infections. *J Clin Lab Anal.* 33, 1–7. doi:10.1002/jcla.22838.

Li, L., Ye, L., Zhang, S., and Meng, H. (2016). Isolation and identification of aerobic bacteria carrying tetracycline and sulfonamide resistance genes obtained from a meat processing plant. *J Food Sci.* 81, M1480–M1484. doi:10.1111/1750-3841.13318.

Liu, C., Fang, Y., Zeng, Y., Lu, J., Sun, Q., Zhou, H., et al. (2020). First report of *OXA-181*-producing *Klebsiella pneumonia*e in China. *Infect Drug Resist*. 13, 995–998. doi:10.2147/IDR.S237793.

Ma, C. (2014). Detection and sequence analysis of correlative virulence genes and drug resistance genes of superiority *Escherichia coli* serotypes isolated from cow mastitis. *Ningxia University*.

Maynard, C., Bekal, S., Sanschagrin, F., Levesque, R. C., Brousseau, R., Masson, L., et al. (2004). Heterogeneity among virulence and antimicrobial resistance gene profiles of extraintestinal *Escherichia coli* isolates of animal and human origin. *J Clin Microbiol.* 42, 5444–5452. doi:10.1128/JCM.42.12.5444-5452.2004.

Ojo, K. K., Ulep, C., Van Kirk, N., Luis, H., Bernardo, M., Leitao, J., et al. (2004). The *mef*(A) gene predominates among seven macrolide resistance genes identified in gram-negative strains representing 13 genera, isolated from healthy Portuguese children. *Antimicrob Agents Chemother.* 48, 3451–3456. doi:10.1128/AAC.48.9.3451-3456.2004.

Osman, K. M., Hassan, H. M., Orabi, A., and Abdelhafez, A. S. T. (2014). Phenotypic, antimicrobial susceptibility profile and virulence factors of *Klebsiella pneumoniae* isolated from buffalo and cow mastitic milk. *Pathog Glob Health.* 108, 191–199. doi:10.1179/2047773214Y.0000000141.

Park, C. H., Robicsek, A., Jacoby, G. A., Sahm, D., and Hooper, D. C. (2006). Prevalence in the United States of *aac(6′)-Ib-cr* encoding a ciprofloxacin-modifying enzyme. *Antimicrob Agents Chemother.* 50, 3953–3955. doi:10.1128/AAC.00915-06.

Poirel, L., Walsh, T. R., Cuvillier, V., and Nordmann, P. (2011). Multiplex PCR for detection of acquired carbapenemase genes. *Diagn Microbiol Infect Dis.* 70, 119–123. doi:10.1016/j.diagmicrobio.2010.12.002.

Rebelo, A. R., Bortolaia, V., Kjeldgaard, J. S., Pedersen, S. K., Leekitcharoenphon, P., Hansen, I. M., et al. (2018). Multiplex PCR for detection of plasmid-mediated colistin resistance determinants, *mcr-1*, *mcr-2,* *mcr-3*, *mcr-4* and *mcr-5* for surveillance purposes. *Eurosurveillance.* 23, 1–11. doi:10.2807/1560-7917.ES.2018.23.6.17-00672.

Riaño, I., Moreno, M. A., Teshager, T., Sáenz, Y., Domínguez, L., and Torres, C. (2006). Detection and characterization of extended-spectrum β-lactamases in *Salmonella enterica* strains of healthy food animals in Spain. *J Antimicrob Chemother.* 58, 844–847. doi:10.1093/jac/dkl337.

Sáenz, Y., Briñas, L., Domínguez, E., Ruiz, J., Zarazaga, M., Vila, J., et al. (2004). Mechanisms of resistance in multiple-antibiotic-resistant *Escherichia coli* strains of human, animal, and food origins. *Antimicrob Agents Chemother.* 48, 3996–4001. doi:10.1128/AAC.48.10.3996-4001.2004.

Tabaran, A., Mihaiu, M., Tăbăran, F., Colobatiu, L., Reget, O., Borzan, M. M., et al. (2017). First study on characterization of virulence and antibiotic resistance genes in verotoxigenic and enterotoxigenic *E. coli* isolated from raw milk and unpasteurized traditional cheeses in Romania. *Folia Microbiol (Praha).* 62, 145–150. doi:10.1007/s12223-016-0481-8.

Veldman, K., Cavaco, L. M., Mevius, D., Battisti, A., Franco, A., Botteldoorn, N., et al. (2011). International collaborative study on the occurrence of plasmid-mediated quinolone resistance in *Salmonella enterica* and *Escherichia coli* isolated from animals, humans, food and the environment in 13 European countries. *J Antimicrob Chemother.* 66, 1278–1286. doi:10.1093/jac/dkr084.

Xu, S., Yang, S., Weng, S., Chen, C, Zhang, W., J, J. (2019). Distribution of serotypes and virulence genes in hypervirulent *Klebsiella pneumoniae* and exploration of molecular markers. *Journal of Microbes and Infection* 14, 338–344.

Yang, F., Deng, B., Liao, W., Wang, P., Chen, P., and Wei, J. (2019). High rate of multiresistant *klebsiella pneumoniae* from human and animal origin. *Infect Drug Resist.* 12, 2729–2737. doi:10.2147/IDR.S219155.

Zhang, Y., Zhao, C., Wang, Q., Wang, X., Chen, H., Li, H., et al. (2016). High prevalence of hypervirulent *Klebsiella pneumoniae* infection in China: Geographic distribution, clinical characteristics, and antimicrobial resistance. *Antimicrob Agents Chemother.* 60, 6115–6120. doi:10.1128/AAC.01127-16.
